# Efficient and stable organic solar cells enabled by multicomponent photoactive layer based on one-pot polymerization

**DOI:** 10.1038/s41467-023-36413-3

**Published:** 2023-02-21

**Authors:** Bin Liu, Huiliang Sun, Jin-Woo Lee, Zhengyan Jiang, Junqin Qiao, Junwei Wang, Jie Yang, Kui Feng, Qiaogan Liao, Mingwei An, Bolin Li, Dongxue Han, Baomin Xu, Hongzhen Lian, Li Niu, Bumjoon J. Kim, Xugang Guo

**Affiliations:** 1 Guangdong Engineering Technology Research Center for Photoelectric Sensing Materials & Devices, Guangzhou Key Laboratory of Sensing Materials & Devices, Center for Advanced Analytical Science, School of Chemistry and Chemical Engineering, Guangzhou University, Guangzhou, 510006 P.R. China; 2 Department of Materials Science and Engineering, Southern University of Science and Technology, Shenzhen, 518055 P.R. China; 3 Department of Chemical and Biomolecular Engineering, Korea Advanced Institute of Science and Technology (KAIST), Daejeon, 34141 Republic of Korea; 4 State Key Laboratory of Analytical Chemistry for Life Science, School of Chemistry & Chemical Engineering and Center of Materials Analysis, Nanjing University, Nanjing, 210023 P.R. China; 5 Songshan Lake Materials Laboratory Dongguan, Guangdong, 523808 P.R. China

**Keywords:** Solar cells, Photovoltaics

## Abstract

Degradation of the kinetically trapped bulk heterojunction film morphology in organic solar cells (OSCs) remains a grand challenge for their practical application. Herein, we demonstrate highly thermally stable OSCs using multicomponent photoactive layer synthesized via a facile one-pot polymerization, which show the advantages of low synthetic cost and simplified device fabrication. The OSCs based on multicomponent photoactive layer deliver a high power conversion efficiency of 11.8% and exhibit excellent device stability for over 1000 h (>80% of their initial efficiency retention), realizing a balance between device efficiency and operational lifetime for OSCs. In-depth opto-electrical and morphological properties characterizations revealed that the dominant PM6-*b*-L15 block polymers with backbone entanglement and the small fraction of PM6 and L15 polymers synergistically contribute to the frozen fine-tuned film morphology and maintain well-balanced charge transport under long-time operation. These findings pave the way towards the development of low-cost and long-term stable OSCs.

## Abstract (web summary)

Degradation of kinetically bulk heterojunction film morphology in organic solar cells is a grand challenge for their practical application. Here, the authors design and synthesise multicomponent photoactive material by facile one-pot polymerization and achieve efficiency of 11.8% and T80 of 1000 h.

## Introduction

Organic solar cells (OSCs) with the bulk heterojunction (BHJ) active layer have drawn wide-spread attention because of their multiple advantages such as high mechanical flexibility, light weight, and semi-transparency^1,2^. Driven by the rapid development of organic photovoltaic materials and device engineering, OSCs have achieved power conversion efficiencies (PCEs) of over 18%^3–7^. Despite the great success in PCE enhancement, the practical applications of OSCs are limited by their low operational stability^8–11^. Of note is the degradation of photoactive layer morphology under various enviromental conditions (i.e. light, oxygen and heat) after long-term exposure, which remains a grand challenge for the commercialization of OSC technology^12–18^.

Highly efficient OSCs require an optimized BHJ morphology with abundant electron donor/electron acceptor (D/A) interfaces for efficient exciton dissociation and bicontinuous D and A domains for charge transport^19–24^. However, the intrinsic instability of the blend photoactive layer commonly occurs during prolonged illumination or thermal aging^25–28^. In particular, an evolution of the blend morphology from the kinetically trapped state to the thermodynamically stable state, such as the aggregation and/or the D/A interface recession, will result in severe degradation of charge dissociation or transfer and unsatisfactory lifetime of OSCs^29–31^. For instance, Martorell^32^ and McGehee^33^ found D and A domain disordering caused by UV light, and Brabec group^34^ reported the major lifetime loss mechanism induced by poor D/A miscibility. Besides, Lidzey et al. demonstrated that thermal annealing above the glass transition temperature of the photoactive layers will also accelerate the device degradation^35,36^. Therefore, it is imperative to develop effective strategies to achieve stable optimized BHJ morphology and thus increase the device stability^37^.

OSCs based on polymer/polymer blends as photoactive layers (all-polymer solar cells, all-PSCs) exhibited higher morphological stability than other kinds of OSCs due to the strong interchain entanglements in all-polymer blend^38–45^. However, their all-polymer BHJ blends suffered from thermodynamically unfavorable miscibility and large-scale phase separation under severe surroundings^46^. To this end, a few studies have demonstrated that the stability issues could be overcome by using well-tailored dual functional single-component photovoltaic materials, in which two or more D and A components are covalently bonded in one material, e.g. molecular dyads, double-cable type polymers and conjugated block copolymers consisting of D and A as blocks^47,48^. These materials can form thermodynamically stable phase-separated domains with appropriate length scales. Thereinto, block copolymers are considered as one of the most promising candidates due to the rapidly increased PCEs for their corresponding OSCs. Nevertheless, the free volumes in the block copolymer networks will lead to the chain segment motion^49^, resulting in difficulty to overcome the trade-off between initial photovoltaic performance and long-term operation stability for OSCs. More importantly, single-component photovoltaic materials suffer from either synthetic challenges or complex purifications.

Herein, we proposed a strategy to develop multicomponent photoactive layers via a facile one-pot polymerization without the complex synthesis and post-treatment, achieving highly efficient and stable OSCs. It should be noted that additional steps and time costs for the polymer purification and device fabrication are obviously reduced in our work. The multicomponent photoactive layer (named S11) is dominated by a block-conjugated polymer PM6-*b*-L15 featuring a donor1-acceptor1-acceptor2-acceptor3 (*d*1-*a*1-*a*2-*a*3) backbone architecture. The multicomponent photoactive layer is synthesized by adding the monomers in sequence during polymerization (for details see Supporting Information), followed by simple flash column chromatography to remove unreacted monomers and small amounts of catalysts. Compared to its analogue S9 with a dominant traditional *d*1-*a*1-*d*2-*a*2 type block polymer, OSCs fabricated from S11 yielded a much higher performance with an optimal PCE approaching 12%, which is enabled by incorporating more electron-deficient building blocks (a.k.a. *a* units) into the polymer backbones. In parallel, the S11-based devices exhibited much superior light and thermal stabilities (maintaining > 80% of their initial PCEs after over 1000 h) to those of the corresponding single-component and two-component photoactive layer-based devices. Through the careful analysis of the opto-electrical and morphological properties, we assume that the block copolymer interlaced networks with backbone entanglements enabled by block polymer PM6-*b*-L15 were formed in the S11 multicomponent system. The small amount of PM6 and L15 polymers in S11 matrix will fill into the nanovoids of the interlaced nanostructures, restricting the chain motion of block copolymers. Meanwhile, the networks formed by PM6-*b*-L15 blocks could in turn suppress the excessive aggregates of PM6 and L15 polymers in the multi-component system. As a result, the favorable frozen film morphology of the networks with well-balanced charge transfer affords an excellent device performance and lifetime prolonging for the OSCs. Hence, this work provides a promising synthetic avenue via one-pot polymerization for the development of the multicomponent photoactive layers in the OSCs with both excellent device efficiency and operational stability.

## Results

### Material properties

**Fig. 1:**
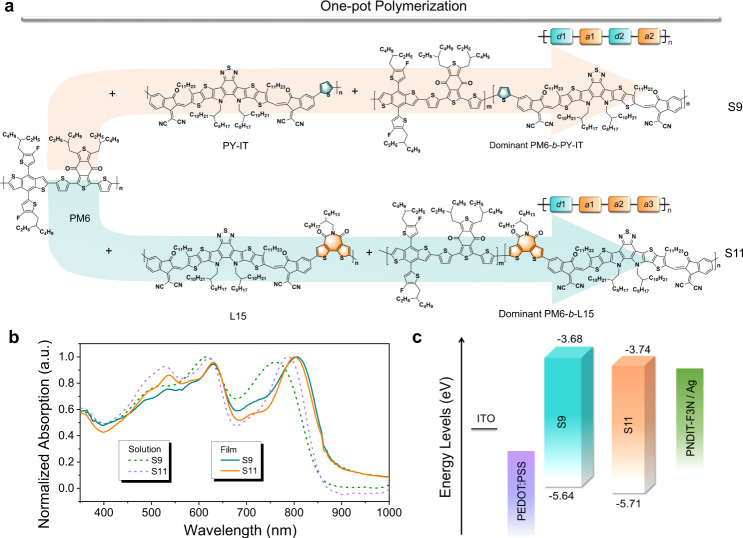
Design, synthesis and opto-electrical properties of multicomponent system S9 and S11. **a** Chemical structures of S9 and S11 synthesized via one-pot polymerization (for details see Supporting Information). **b** UV-vis absorption spectra of S9 and S11 in chloroform solution and film. **c** Energy level diagrams of multicomponent systems and electrodes used in the OSCs.

The multicomponent photoactive layers were facilely synthesized via addition polymerization in one pot^50,51^. The expected chemical structures of main composition in the multicomponent photoactive layers are shown in Fig. 1a. The monomers *d*1, *d*2 and *a*1 are commercially purchased, and the monomers *a*2 and *a*3 are synthesized according to our previously reported protocols^52^ (Supplementary Figs. 1–5). The multicomponent photoactive layers of S9 and S11 are developed in a similar way for block polymers by adding the monomers in sequence. The photographic images showing the progress of one-pot polymerization are depicted in Supplementary Fig. 6. The resulting polymers were treated with simple flash column chromatography for removing the residual palladium catalysts and unreacted monomers. Unlike the time-consuming post-treatment such as Soxhlet extraction, the procedures for material synthesis are evidently simplified. For the synthesis of the conjugated diblock or triblock polymers, a small amount of low *M*_n_ oligmers or polymer blocks (PM6 and L15 or PY-IT blocks in our case) are indeed included except the majority of ambipolar block copolymers (PM6-*b*-PY-IT or PM6-*b*-L15 here) even in quasi-living polymerization and self-switchable alternating copolymerization for conjugated block polymers^53,54^. Therefore, the obtained multicomponent polymers S11 (or S9) from the one-pot synthesis method may contain some of PM6, L15 (or PY-IT), and PM6-*b*-L15 (or PM6-*b*-PY-IT) due to deactivated polymerization sites, but the block copolymers are in majority^50,51^. For clear comparison, the purified block copolymers of PM6-*b*-PY-IT and PM6-*b*-L15 were also obtained via the reported post-processing method of column chromatography and Soxhlet extraction^46,55^. The number-average molecular weight (*M*_n_) and polydispersity index (PDI) of the intermediate PM6 block and target block materials measured by gel permeation chromatography (GPC) are summarized in Supplementary Figs. 7–9 and Supplementary Table 1. The *M*_n_ of the PM6 block was 6.9 kDa with a PDI of 1.85 while the *M*_n_ values of S9 and S11 were 16.3 kDa and 18.6 kDa with PDIs of 2.69 and 2.71, respectively, which confirms that the target products were successfully synthesized. Furthermore, the structural characteristics of PM6 block and the synthesized materials were determined by the hydrogen nuclear magnetic resonance (^1^H NMR) (Supplementary Figs. 10–15). The molar ratios of PM6 and L15 (or PY-IT) units in the block copolymers and multicomponent materials were estimated by X-ray photoelectron spectroscopy (XPS) and energy-dispersive X-ray spectroscopy (EDX)^56^. On the basis of the XPS spectra of S9 and S11 in Supplementary Fig. 16, the ratios of F and N concentration are 1:2.70 and 1:3.03, demonstrating that the ratios of PM6 block:PY-IT block and PM6 block:L15 block in the final systems are around 1:0.92 and 1:0.91, respectively. In addition, the molar ratios of the PM6 block:PY-IT block and PM6 block:L15 block were determined to be ~1:0.89 and 1:0.91 in S9 and S11 systems from the EDX data (Supplementary Fig. 17 and Supplementary Table 2), respectively, agreeing well with the XPS results. Moreover, we have further determined the multicomponent composition and structure of S11 via the available technologies such as sequential purification, XPS, and the elemental analysis (EA) (for details see Supporting Information, Supplementary Fig. 18). The mass contents of the dissociative (or residual) PM6 polymer and L15 polymer in S11 were determined to be 1.94% and 5.29% (Supplementary Tables 3 and 4). Thus, our S11 materials are multicomponent mixtures including PM6 polymer (~2%), L15 polymer (~5%) and PM6-*b*-L15 block polymer (~93%).

Furthermore, the corresponding UV-vis absorption spectra of S9 and S11 in chloroform solutions and thin films are presented in Fig. 1b. Both films exhibit red-shifted absorption compared to their solutions due to the improved molecular ordering from solution to solid film. As expected, S11 film shows a broad absorption in the range of 400–900 nm with a narrow *E*_*g*_ of ~1.38 eV and contains the obvious features of the corresponding PM6 and L15 moiety, e.g., three distinct absorption peaks at 536, 631, and 802 nm, and no new absorption peaks were recorded. By contrast, S9 film exhibits similar absorption ranges but with lower absorption in 450–600 nm and slightly higher absorption in the wavelength range of 650-800 nm. The LUMO levels (*E*_LUMO_) and HOMO levels (*E*_HOMO_) were probed through cyclic voltammetry (CV) measurements. As shown in Fig. 1c and Supplementary Table 5, from S9 to S11, the *E*_LUMO_/*E*_HOMO_s were altered from −3.68/−5.64 to −3.74/−5.71 eV. It is found that the frontier molecule orbital energy levels are slightly increased due to the replacement of thiophene (*d*2) by bithiophene imide (*a*3) as co-unit. As we mentioned above, the multicomponent polymers S11 (or S9) from the one-pot method via simple post processing contained a small fraction of PM6, L15 (or PY-IT), but the block copolymers were indeed in majority. As shown in Supplementary Fig. 19, the multiple oxidation and reduction states in CV plots have also corroborated the multicomponent nature.

### Photovoltaic performances

**Fig. 2:**
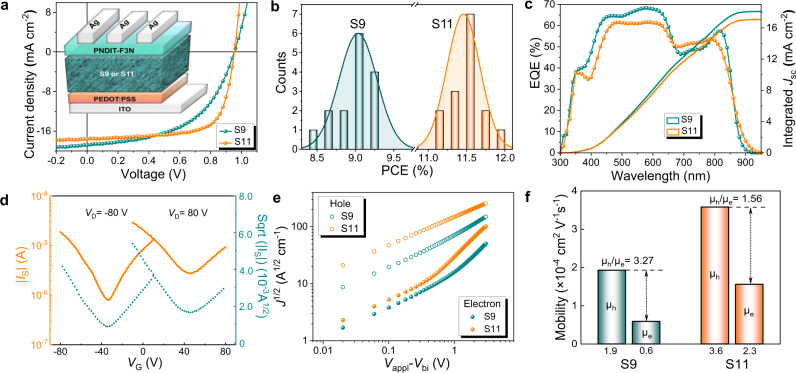
Photovoltaic properties of OSCs. **a** *J–V* characteristics for OSCs and the corresponding device structure used in this work. **b** Histograms of the PCE counts for 15 individual devices. **c** EQE spectra and integrated *J*_sc_ for devices based on S9 and S11. **d** Transfer characteristics of the S11-based top-gate/bottom-contact (TGBC) organic field-effect transistors (OFETs). **e** *J-V* curves of the hole-only and electron-only devices containing the optimal active layer in dark. **f** Mobility ratios for the corresponding devices.

To evaluate the photovoltaic performance of multicomponent photoactive layers S9 and S11, OSCs were fabricated with a device structure of ITO/PEDOT:PSS/S9 (or S11)/PNDIT-F3N/Ag. The electron transporting layer PNDIT-F3N used in this work was measured to have a *M*_n_ of 4.0 kDa with a PDI of 1.46^57^ (Supplementary Fig. 20). The devices based on the purified block copolymer of PM6-*b*-L15 were also prepared as control. As illustrated in Fig. 2a and summarized in Table 1, the S11-based OSCs yield an optimal PCE of 11.78 % with a fill factor (FF) of 70.11%, an open-circuit voltage (*V*_oc_) of 0.95 V and a short-circuit currenty (*J*_sc_) of 17.62 mA cm^−2^. In comparison, the S9-based devices show a much lower PCE of 9.34 % mainly due to the low FF value of 52.15%. In addition, the average PCE values reach 9.05 % and 11.43 % for the S9- and S11-based OSCs, respectively. The PCE values of the S11-based OSCs obtained from 15 different devices show narrow distribution (Fig. 2b), demonstrating good device reproducibility. Moreover, the repeatability of photovoltaic materials is also critical. To this end, to probe into the effects of molecular weight of donor and acceptor blocks on device performances, various batches of S11 are synthesized by changing the reaction time of PM6 (S11-D1, S11-D2 and S11-D3) and L15 blocks (S11-A1, S11-A2, and S11-A3) (Supplementary Figs. 21–26 and Supplementary Tables 6–9). It was found that the variable polymerization time of PM6 donor blocks can exert a significant influence on the material properties and the device performances. As the polymerization time of PM6 block prolonged, the *J*_sc_ and FF values increased from *J*_*sc*_ = 11.36 mA cm^−2^ and FF = 56.47% for the S11-D1-based device to *J*_*sc*_ = 17.51 mA cm^−2^ and FF = 70.32 % for the S11-D2-based one. As a result, the S11-D2-based OSCs achieved the highest PCE of 11.72% among three multicomponent systems. By contrast, almost little impact on the OSC efficiencies (11.76% for S11-A1, 11.53% for S11-A2 and 11.06% for S11-A3) was observed when the polymerization periods of L15 acceptor blocks were greatly changed but with the fixed optimal reaction time for PM6 blocks (the details can be found in the Supporting Information). Furthermore, the S11-based device with 0.8 cm^2^ area yielded a PCE of 11.02 % while the analogue with 1.0 cm^2^ achieved a PCE of 10.80 % (Supplementary Fig. 27), demonstrating its feasibility in large area preparation. It should also be noted that the PCE of 14.3 % was attained for our purified PM6-*b*-L15 system, which is among the highest PCE values for single component-based OSCs^46^ (Supplementary Figs. 28, 29 and Supplementary Table 10). The external quantum efficiency (EQE) spectra and integrated *J*_sc_s of the optimized OSCs are shown in Fig. 2c. The S11-based devices exhibit stronger photon response from 650 to 800 nm but weaker one from 400 to 600 nm than the S9-based OSCs, consistent with the absorption properties of S9 and S11. The *J*_sc_s integrated from the EQE spectra are 18.09 and 17.06 mA cm^−2^ for the S9 and S11-based OSCs, respectively, which are close to *J*_sc_ values from *J-V* curves with a mismatching below 5%.

**Table 1:** Photovoltaic performance parameters of the OSCs incorporating S9 and S11 photoactive layers under the illumination of AM 1.5 G,100 mW cm^−2^

Photoactive layer^a^	*V*_oc_ (V)	*J*_sc_ (mA cm^−^^2^)	Cal *J*_sc_ (mA cm^−^^2^)^b^	FF (%)	PCE (%)
S9	0.95 (0.95 ± 0.01)	18.79 (18.53 ± 0.27)	18.09	52.15 (51.38 ± 0.68)	9.34 (9.05 ± 0.29)
S11	0.95 (0.95 ± 0.01)	17.62 (17.38 ± 0.24)	17.06	70.11 (69.26 ± 0.86)	11.78 (11.43 ± 0.35)

Data in parentheses are average values with standard deviation from 15 devices.

^a^The device area: 4.5 mm^2^.

^b^Integrated current density obtained from EQE spectra.

### Charge dissociation, transport and collection mechanism

To evaluate the charge transport properties of S9 and S11, top-gate/bottom-contact (TGBC) organic field-effect transistors (OFETs) were then fabricated^55^. As shown in Fig. 2d, Supplementary Figs. 30, 31 and Supplementary Table 11, both multicomponent photoactive layers exhibit ambipolar transport characteristics. After annealing at elevated temperature to optimize the morphology, S11 shows hole mobility (*μ*_h,OFET_) of 1.43 × 10^−2^ cm^2^  V^−1^ s^−1^ and electron mobility (*μ*_e,OFET_) of 6.19 × 10^−3^ cm^2^ V^−1^ s^−1^, which is much higher than S9 with a *μ*_h,OFET_ of 5.90 × 10^−3^ cm^2^ V^−1^ s^−1^ and a *μ*_e,OFET_ of 8.65 × 10^−4^ cm^2^ V^−1^ s^−1^, suggesting p-type dominant transport behavior. Please note that the *μ*_e,OFET_ of S11 is more than an order of magnitude higher than that of S9, corroborating a larger fraction of acceptor units in S11^58,59^. The trend correlates well with that of the results obtained from the space-charge limited current (SCLC) method (Fig. 2e and Supplementary Table 12). The SCLC hole and electron mobilities (*μ*_h,SCLC_s and *μ*_e,SCLC_s) are measured to be 1.93 × 10^−4^ and 0.59 × 10^−4^ cm^2^ V^−1^ s^−1^ for S9, and 3.58 × 10^−4^ and 2.29 × 10^−4^ cm^2^ V^−1^ s^−1^ for S11, respectively. The more balanced mobilities with a *μ*_h,SCLC_/*μ*_e,SCLC_ ratio of 1.56 were obtained in the S11-based devices (Fig. 2f), which explains the higher FF values.

The photocurrent density (*J*_ph_) versus the effective applied voltage (*V*_eff_) and the relationship between *J*_sc_, *V*_oc_ and light intensity (*P*_light_) were probed to understand the charge generation and dissociation process. As depicted in Supplementary Fig. 32a, the *J*_ph_s reach the saturation value (*J*_sat_) under high *V*_eff_ (>1.0 V), indicating that each photo-generated exciton dissociated into free electron and hole. The calculated maximum exciton generation rates (*G*_max_) of the devices are included in Supplementary Table 13. The S11-based device achieved slightly higher *G*_max_ value (1.20 × 10^28 ^m^−3^ s^−1^) than the S9-based analogue (1.16 × 10^28 ^m^−3^ s^−1^). The exciton dissociation probability (*P*_diss_) can be obtained from the ratio between *J*_ph_^*a*^ (*J*_ph_ under short circuit condition) and *J*_sat_, while the the charge collection efficiency (*P*_coll_) from the ratio between *J*_ph_^*b*^ (*J*_ph_ under the maximal power output condition) and *J*_sat_. Hence, the *P*_diss_ and *P*_coll_ for the S9-based OSCs are 86.0% and 74.8%, whereas the corresponding values are 91.9% and 83.2% for S11-based ones (Supplementary Table 14), indicating a significantly improved exciton dissociation efficiency in the S11-based OSCs. Photoluminescence (PL) quenching measurements were subsequently conducted (Supplementary Fig. 32b). The S11 film showed a higher PL quenching efficiency of 88%, indicating more efficient charge transfer. In addition, to better understand the charge recombination process, the dependence of *J–V* characteristics on light intensity (*P*_light_) was studied. The exponential factor (*α*) close to 1 demonstates negligible bimolecular recombination according to the power-law relevance *J*_sc_∝*P*_*light*_^*α*^ ^60^. As presented in Supplementary Fig. 33, the S11-processed OSC showed a higher *α* value of 0.961 than the S9-based one (0.852). Therefore, free charges generated in the S11-based devices can be more efficiently transported to the electrodes than those in the S9-based cells. These results demonstrated that the S11-based devices exhibit better exciton dissociation and charge collection, corroborating their higher photovoltaic performance parameters than the S9-based analogues.

### Photoactive layer morphology

**Fig. 3:**
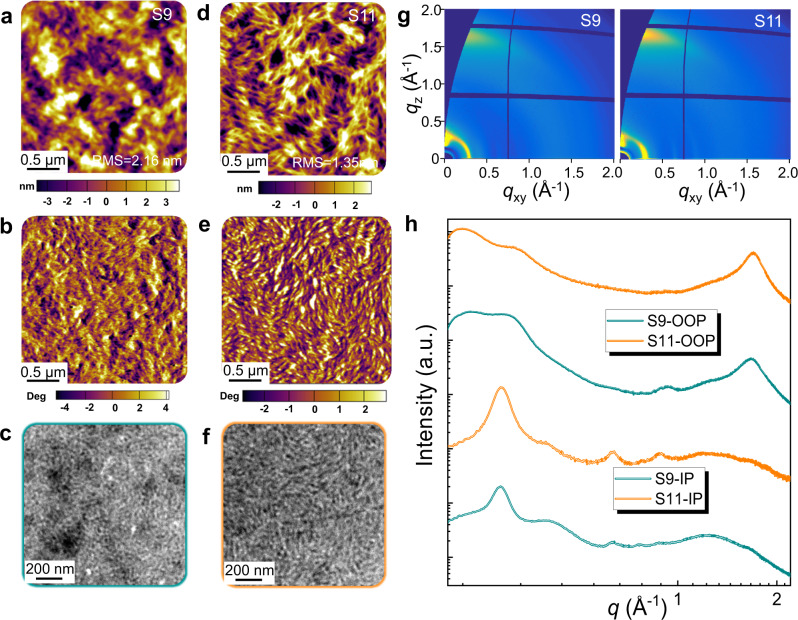
Morphological characteristics of the films. **a**, **b** AFM height, **c**, **d** phase and **e**, **f** TEM images of S9 and S11 films. **g** 2D GIWAXS patterns and **h** scattering profiles of out-of-plane and in-plane for their corresponding films.

Atomic force microscopy (AFM) and transmission electron microscopy (TEM) measurements were firstly performed to investigate the film morphological features. As shown in Fig. 3a, b, the root-mean-square (RMS) roughness value of S9 film is 2.2 nm, which is about two fold larger than that of S11 film. Interestingly, the AFM phase (Fig. 3c, d) and TEM images (Fig. 3e, f) of the S11-based film show distinctive fibrillar textures, which should be beneficial to charge generation and transport in the corresponding OSCs. Furthermore, molecular ordering in photoactive layers was investigated through grazing incidence wide angle x-ray scattering (GIWAXS) measurements (Fig. 3g, h and Supplementary Table 15). The S11 film exhibits significantly stronger and sharper scattering peaks in the in-plane (IP) direction (*q*_xy_ = 0.29 Å^−1^) and the out-of-plane (OOP) direction (*q*_z_ = 1.66 Å^−1^) compared to the S9 film. To explain this difference more quantitatively, we compared the crystal coherence lengths (*L*_c_s) estimated from the Scherrer equation^15^. The S11-based photoactive film shows increased crystal sizes in both lamellar (20.4 vs.19.9 nm) and π–π stackings (3.85 vs. 2.83 nm) in comparison to the S9 film, leading to enhanced charge transport properties and better performance in the S11-based OSCs^61^. Furthermore, the photo-induced force microscopy (PiFM) was conducted to better understand the interpenetrating networks for S9 and S11 systems^62,63^. The PM6:L15 blend film was also prepared for a better comparison. As depicted in Supplementary Fig. 34, the PiFM pattern of PM6:L15 blend film shows uniform but unclear phase separation with a few fibrous aggregates. In contrast, the PiFM images of both S9 and S11 display more pronounced fibrillar nanonetwork. Especially, the longer backbone and more favorable entanglement of S9 and S11 than that of PM6:L15 blend can be observed from the magnified PiFM images with scale bar at 100 nm (Supplementary Fig. 35), confirming the bi-continuous network structures in S9 and S11 multicomponent systems.

### Device stability

**Fig. 4:**
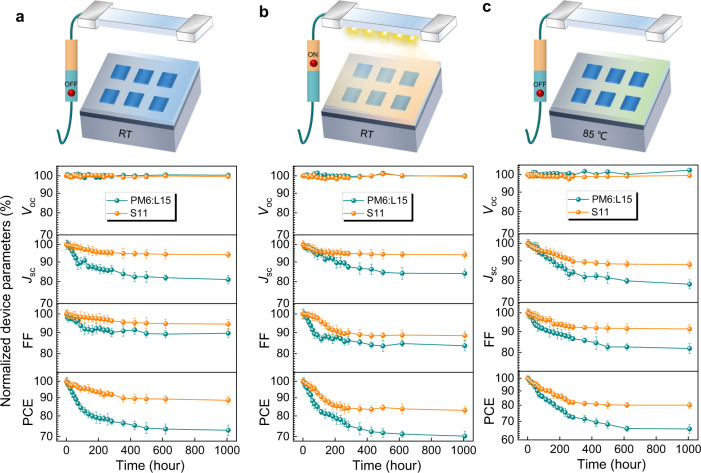
Shelf-life, light and thermal stability. Evolution of photovoltaic parameters of the optimized two-component system PM6:L15- and multicomponent system S11-based OSCs in nitrogen atmosphere for up to 1008 h storage: **a** under room temperature and dark condition, **b** under continuous illumination condition, **c** under 85 °C heated condition.

The operational stabilities of the multicomponent photoactive layer S11-based OSCs were monitored by tracking the *J-V* characteristics under dark, illumination, and thermal aging conditions for over 1000 h, respectively. For a better comparison, we also measured the corresponding stabilities of two-component PM6:L15-based devices as the control. Please note that the OSCs based on the PM6:L15 blend were fabricated with the donor: acceptor weight ratio of 1:1.2, achieving a PCE of 15.38%, which is consistent with the previous report (15.20%)^52^. To probe the differences between fresh PM6:L15 binary blend and S11 multicomponent system, the AFM, TEM, and GIWAXS characterizations were employed to compare the morphology, and the transient absorption spectroscopy (TAS) was correspondingly performed to better understand the hole transfer kinetics in both systems. The detailed discussion is included in Supporting Information (Supplementary Figs. 36–37 and Supplementary Table 16). The periodically monitored photovoltaic performances are depicted in Fig. 4, and the changes in the *J-V* curves and the parameters are depicted in Supplementary Fig. 38 and Supplementary Tables 17–22. Regarding the shelf-life stability, the S11-based devices exhibit a slight PCE decrease and retain ~90% of original PCEs after 1000 h storage at room temperature (RT), which mainly stems from the burn-in degradation of electrodes and interfaces^55^. Nevertheless, the analogs with PM6:L15 suffer from ~28% degradation of PCEs during the same intervals. Likewise, 83% of its initial efficiencies were preserved in the multicomponent photoactive layer S11-based OSCs, while the performance for the two-component devices drops to 70 % after light-soaking with the irradiation intensity of 100 mW cm^−2^ white LED for more than 1000 h. Furthermore, unencapsulated devices under continuous thermal stress of 85 °C demonstrate a similar trend in these two systems. The two-component-based OSCs show dramatically decreased PCE with only 65% of the initial PCEs retained. On the contrary, the multicomponent S11-based OSCs exhibit excellent thermal stability, maintaining 80% of its original PCEs after 1000 h thermal aging. It is found that the multicomponent S11-based OSCs show more stable *J*_sc_ and FF values than the binary ones during the same period of device aging. Hence, our multicomponent OSCs reach a better balance between the PCEs and device stability compared to the single-component OSCs (Supplementary Fig. 39). These results unravel the significant effects on mitigating material property and film morphology degradation via the facile one-pot polymerization^64–66^.

**Fig. 5:**
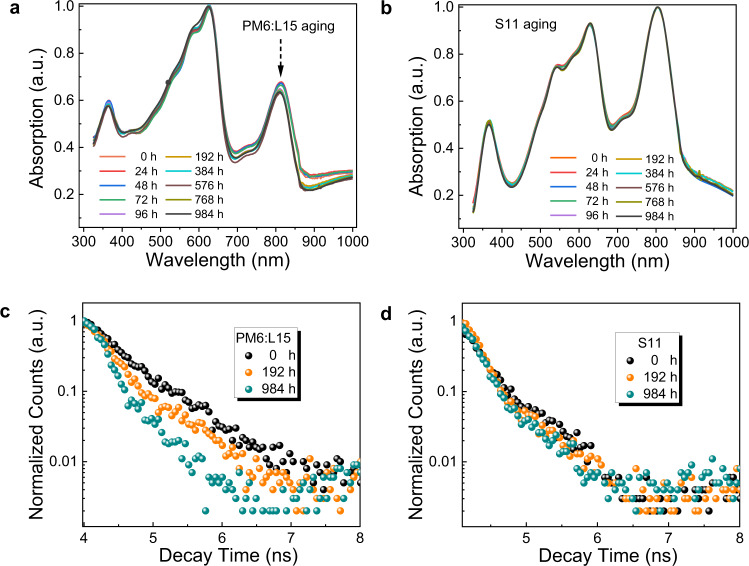
Absorption property and fluorescence lifetime as a function of aging time. Normalized UV-vis absorption spectra of aged **a** two-component system PM6:L15 and **b** multicomponent system S11 films with different heating duration. Normalized TRPL decay curves of **c** PM6:L15 and **d** S11 films as a function of storage time.

The PM6:L15 and S11-based OSCs show distinct degrees of efficiency drops during the thermal aging, which triggers our interest to focus on the thermal stability in terms of property and morphology evolution for the corresponding photoactive layers. The UV-vis absorption and time-resolved photoluminescence (TRPL) aging tests during 984 h heating (at 85 °C) were tracked at regular intervals. The UV-vis absorption spectra of both photoactive layers before and after heating are shown in Fig. 5a, b. For the PM6:L15 blend film, absorption intensity gradually decreases with prolonged heating time, suggesting a low thermal stability of the two-component film. In contrast, S11 film shows negligible change of absorption spectrum after ~1000 h heating aging. This result indicates that S11 can well retain its initial microstrcuture, which accounts for the enhanced thermal stability of OSCs. Besides, the normalized TRPL decay curves of PM6:L15 and S11 films as a function of storage time are depicted in Fig. 5c, d. The fluorescence lifetime (τ) of the PM6:L15 photoactive layer is calculated to be 540 ps for 0 h and 270 ps for 984 h. The shortened lifetime is not conducive to the extion diffusion in the D/A interfaces, leading to a sharp decline of its device performance. Impressively, the multicomponent S11 film holds almost unchanged fluorescence lifetime with a τ of 360 ps for 0 h and τ of 310 ps for 984 h, ensuring efficient exciton diffusion between D/A interfaces, and, thereby yielding more stable *J*_sc_ and FF^67,68^.

**Fig. 6:**
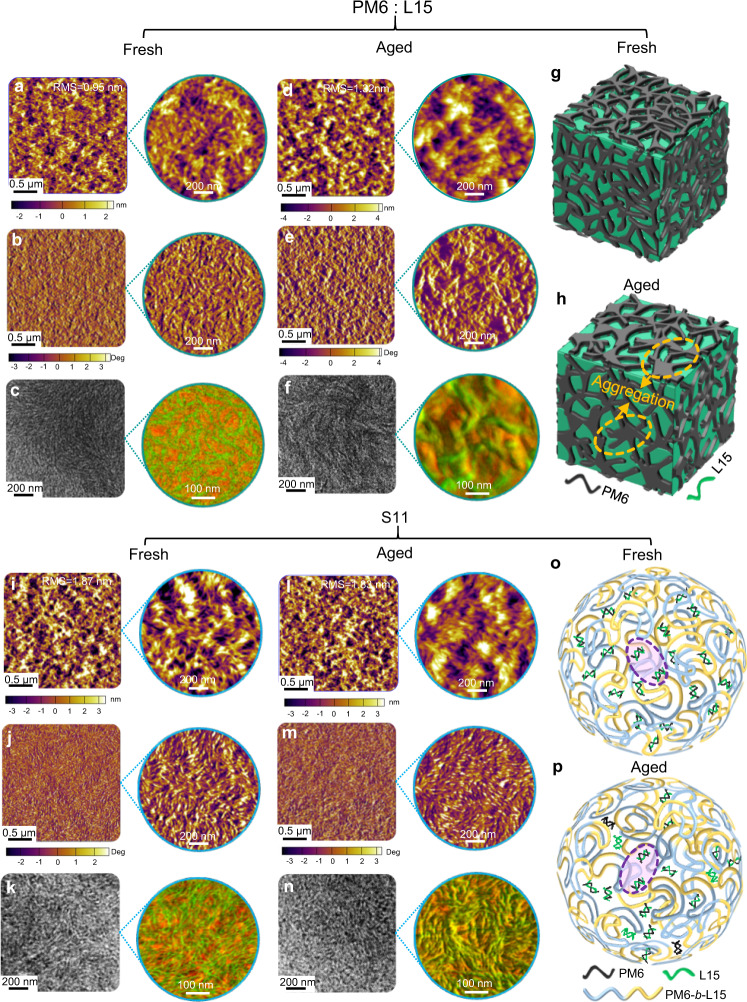
Morphology evolution during the aging test. The OSCs based on PM6:L15 and S11 active layers under continuous heating of 984 h: **a**, **d**, **i**, **l** AFM height, **b**, **e**, **j**, **m** phase and **c**, **f**, **k**, **n** TEM and combined PiFM images for two-component system PM6:L15 and multicomponent system S11 films before and after aging, respectively. Schematic illustration of morphology changes for **g**, **h** two-component system and **o**, **p** multicomponent system before and after thermal aging, respectively.

To gain insights into the correlation between exciton diffusion decay and BHJ microstructure changes, the AFM and TEM images for thin films were obtained under 984 h continuous thermal aging (Fig. 6a–f, i–n). All the fresh films based on PM6:L15 and S11 exhibit distinctive fibrous nanostructures^69^. After over 1000 h heating, RMS roughness of the PM6:L15 blend significantly increased from 0.95 to 1.32 nm, and excessive large-sized polymer aggregates emerged in the AFM images for the aged two-component system. In contrast, the multi-component system showed no change in the RMS roughness and the same BHJ homogeneity after annealing. The negligible change has been further confirmed by TEM measurement, which proves that the presence of block polymers can effectively suppress the further aggregation of a handful of D and A in S11. To reveal the morphology stability of our multi-component system more intuitively, the PiFM technique was accordingly conducted. After 716 h thermal annealing, the multicomponent S11 film retains distinguishable phase separation and distinctive interpenetrating nanonetwork of the PM6-*b*-L15 block copolymers with a small fraction of PM6 and L15 surrounding the block copolymer domains, which facilitates the exciton dissociation and charge transport^70^. In contrast, the PM6:L15 blend film showed larger donor and acceptor domains with oversized phase separation (Supplementary Fig. 40). Therefore, segregated domains with decreased D/A interfaces led to seriously deteriorated exciton dissociation, which was confirmed by the fluorescence decay dynamics (Fig. 6g, h). Consequently, a rapid drop in device efficiency was observed in the aged two-component film.

**Fig. 7:**
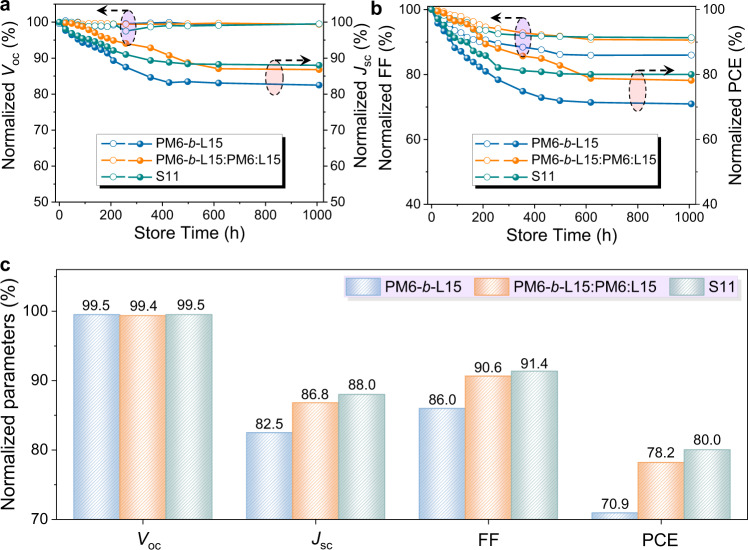
The Comparison of thermal stability. Evolution of **a** *V*_oc_, *J*_sc_, and **b** FF, PCE **c** retained photovoltaic parameters of the optimized single component system PM6-*b*-L15-, the ternary system PM6-*b*-L15:PM6:L15- and the multi-component system S11-based OSCs under 85 °C heated condition.

The stability of the purified PM6-*b*-L15-based devices was also periodically monitored and founded to be far lower than that of our multicomponent system after 1000 h accelerated thermal aging (71% *vs*. 80% maintained, Fig. 7). We considered it is mainly due to morphological instability caused by the chain motion through the free volumes between the block copolymer networks in single PM6-*b*-L15 system^49^. Thus, the contact angle analysis was then implemented to investigate the possible intermolecular interactions in the S11 multicomponent system (Supplementary Fig. 41). The calculated Flory-Huggins interaction parameter (χ) of PM6:PM6-*b*-L15 is very close to that of L15:PM6-*b*-L15 (Supplementary Table 23), indicating a good miscibility for these combinations^71^. Therefore, the PM6 and L15 polymer tended to interact with PM6 block and L15 block of PM6-*b*-L15 backbones in S11 system, respectively. Driven by these favorable interactions, the individual PM6 or L15 polymers can easily fill into the nanovoids of the PM6-*b*-L15 block copolymer interlaced networks, restricting the molecular motion of block copolymers^56^. Based on the above considerations, we assume that the abundant block polymers with long *d*1-*a*1-*a*2-*a*3 backbones firstly tend to self-assemble and form fibrillar nanonetwork structures, freezing the film morphology by embeding the interconnected D and A components into these dense networks (Fig. 6o). Under continuous thermal annealing, the presence of block polymer networks can effectively suppress the migration of D and A matrix (Fig. 6p). Meanwhile, the small amount of residual PM6 and L15 polymers in S11 system can favorably fill free volumes of the entangled block copolymer backbones, thus achieving more compact molecular packing and enhanced morphology stability even under continuous thermal annealing conditions^56^ and, therefore, realizing the efficient charge transfer in the multicomponent system^72–74^. With the synergistic effects of these two factors, reduced charge recombination and facilitated exciton dissociation can be sustained during the aging. Hence, the presence of dominant block polymer networks and small fraction of PM6 and L15 polymers in S11 can affect both the surface and interface characteristics of photoactive layers, and improve the long-term stability of OSCs in a prolonged aging period. To further confirm the assumption, a small amount of polymers PM6 (2%) and L15 (5%) polymers were intentionally incorporated into the purified block copolymer PM6-*b*-L15 as the multicomponent active layer with the exact composition and structure to fabricate the OSC devices. The stability results after 1000 h accelerated thermal aging for the PM6-*b*-L15:PM6:L15 system were comparable to the S11 system prepared by our one-pot method (78% *vs*. 80% retained, Fig. 7). Apparently, our one-pot method is simpler and lower-cost for the device fabrication than the traditional multicomponent system. This result highlights the feasibility of one-pot method to boost the long-term stability of the OSC devices.

## Discussion

In summary, we demonstrated an effective molecular strategy based on an one-pot polymerization for improving the stability of OSCs, reducing synthetic cost and simplifying the device fabrication procedure. The multicomponent photoactive layer S11 is developed in a similar way for block polymers by adding the monomers in sequence, but only purified by simple flash column chromatography to remove small amounts of residual catalyst and monomers without complex post-treatment. The dominant block polymer component in the S11 features a *d*1-*a*1-*a*2-*a*3 backbone architecture. Compared to its analogue polymer with an alternative *d*-*a* backbone structure, incorporating more A unit into the polymer enables higher and more balanced electron and hole mobilities, achieving a decent PCE of 11.8% for the OSCs. More importantly, the multicomponent S11-based OSCs exhibit much more superior device stability than the corresponding two-component ones and the reported single-component OSCs, which endows a better trade-off between PCE and lifetime compared to single-component OSCs, i.e. maintaining > 80% of their initial PCEs even after continuous illumination and thermal annealing for over 1000 h. The systematic investigations on the opto-electrical and morphological evolution as a function of aging time were performed. We assume the majority of block copolymer interlaced networks and a small amount of PM6 and L15 polymers contribute to the excellent stability: (i) the formation of excessive aggregates in the photoactive layer is effectively suppressed while maintaining fibrillar percolations in the D and A domains enabled by the network nanostructures; (ii) the chain backbone motion is significantly restricted by the individual PM6 or L15 polymers packed into the nanovoids of the block copolymer interpenetrated networks. Moreover, the physically mixed PM6:L15:PM6-*b*-L15 multiple components with the exact composition were established to further prove our assumption. The stability results achieved in the PM6:L15:PM6-*b*-L15 multicomponent system were also comparable to the S11 system prepared by this one-pot method. Our one-pot method is simpler and cost-effective for device fabrication, and this work provides an important and practicable strategy to develop highly efficient and operationally stable OSCs toward industrial viability.

## Methods

### Materials

All chemicals and reagents are commercially purchased from vendors and are used without further purification unless otherwise stated. Anhydrous toluene is distilled from Na/benzophenone under argon. The synthesis of Compound a (Supporting Information) is based on the previously reported procedures^52^. Compound IC-Br (Supplementary Fig. 2) is purchased from Hyperchemical, Inc. Unless otherwise stated, all reactions are performed in an inert atmosphere using standard Schlenk-line techniques.

### Fabrication of the OSCs

OSCs with a conventional structure of ITO/PEDOT: PSS/active layers/PNDIT-F3N/Ag are fabricated. The pre-cleaned ITO-coated glass with a sheet resistance of 12 Ω sq^−1^ is used as the substrate and UV-ozone (BZS250GF-TC, HWOTECH, Shenzhen) treatment for 15 min before utilization. PEDOT: PSS (Clevios P VP A1 4083) was spin-coated onto the UV-ozone-treated ITO substrates at 3000 rpm for 40 s and then annealed at 150 °C for 15 min, forming a ~35 nm film. For S9- and S11-based OSCs, the total concentration was fixed at 16 mg mL^−1^, and the films were obtained by spin coating solutions of the polymers in CF containing 1-chloronaphthalene (1-CN, 4 vol%). The solutions were stirred at room temperature for 6 h and heated at 45 °C for about 1 h before use. In addition, the control devices based on PM6:L15 blend processed with CF solvent were also fabricated for comparison. The photoactive layers with an optimal thickness of 100~110 nm are annealed at 100 °C for 5 min. Then PNDIT-F3N dissolved in methanol at the concentration of 1.0 mg mL^−1^ was spin-coated at 3500 rpm for 40 s on the photoactive layers. 100 nm Ag is sequentially deposited on the photoactive layers to complete the devices via thermal evaporation (*ca*. 1 × 10^−5 ^Pa).

### Characterization of the OSCs

All current-voltage *(J-V*) characteristics of the devices were measured under simulated AM1.5 G irradiation (100 mW/cm^2^) using a Xe lamp-based SS-F5-3A Solar Simulator (Enli Technology, Inc.). A Xe lamp equipped with an AM1.5 G filter was used as the white light source. The light intensity was controlled with an NREL-calibrated Si solar cell with a KG-5 filter. The external quantum efficiency (EQE) was measured by a QE-R3011 measurement system (Enli Technology, Inc.).

### AFM measurements

AFM measurements of two- or multicomponent photoactive layers were conducted using a Dimension Icon Scanning Probe Microscope (Asylum Research, MFP-3D-Stand Alone) in the tapping mode.

### TEM measurements

TEM micrographs were obtained on a Tecnai G2 F30 microscope operating at 300 kV.

### GIWAXS measurements

GIWAXS measurements were carried out at the PLS-II 9 A U-SAXS beamline of Pohang Accelerator Laboratory, Korea.

### Reporting Summary

Further information on research design is available in the Nature Portfolio Reporting Summary linked to this article.

## Supplementary information

Supplementary Information

Solar Cells Reporting Summary

## Data Availability

Source data are provided with this paper. All data generated or analyzed during this study are included in the Supplementary Information/Source Data file. The data that support the findings of this study are available from the corresponding author upon request. Source data are provided with this paper.

## Source data

Source Data

## References

[1] Heeger AJ (2014). 25th anniversary article: bulk heterojunction solar cells: understanding the mechanism of operation. Adv. Mater..

[2] Zuo L (2022). Dilution effect for highly efficient multiple-component organic solar cells. Nat. Nanotechnol..

[3] Zhan L (2021). Layer-by-layer processed ternary organic photovoltaics with efficiency over 18%. Adv. Mater..

[4] Zhu C (2020). Tuning the electron-deficient core of a non-fullerene acceptor to achieve over 17% efficiency in a single-junction organic solar cell. Energy Environ. Sci..

[5] Lin Y (2020). Self-assembled monolayer enables hole transport layer-free organic solar cells with 18% efficiency and improved operational stability. ACS Energy Lett..

[6] Li C (2021). Non-fullerene acceptors with branched side chains and improved molecular packing to exceed 18% efficiency in organic solar cells. Nat. Energy.

[7] Wang Z (2020). Thermodynamic properties and molecular packing explain performance and processing procedures of three D18: NFA organic solar cells. Adv. Mater..

[8] Mateker WR, McGehee MD (2017). Progress in understanding degradation mechanisms and improving stability in organic photovoltaics. Adv. Mater..

[9] Zhang Y (2018). Thermally stable all-polymer solar cells with high tolerance on blend ratios. Adv. Energy Mater..

[10] Zhang C (2019). A top-down strategy identifying molecular phase stabilizers to overcome microstructure instabilities in organic solar cells. Energy Environ. Sci..

[11] Bai Q (2022). Recent progress in low-cost noncovalently fused-ring electron acceptors for organic solar cells. Aggregate.

[12] Yang W (2020). Simultaneous enhanced efficiency and thermal stability in organic solar cells from a polymer acceptor additive. Nat. Commun..

[13] Jørgensen M (2012). Stability of polymer solar cells. Adv. Mater..

[14] Kang H (2016). Bulk-heterojunction organic solar cells: Five core technologies for their commercialization. Adv. Mater..

[15] Liu B (2021). Achieving highly efficient all-polymer solar cells by green-solvent-processing under ambient atmosphere. Energy Environ. Sci..

[16] Janssen RA, Nelson J (2013). Factors limiting device efficiency in organic photovoltaics. Adv. Mater..

[17] Liao Q (2022). Highly stable organic solar cells based on an ultraviolet-resistant cathode interfacial layer. CCS Chem..

[18] Li Y (2018). Near-infrared ternary tandem solar cells. Adv. Mater..

[19] Gurney RS, Lidzey DG, Wang T (2019). A review of non-fullerene polymer solar cells: from device physics to morphology control. Rep. Prog. Phys..

[20] Ye L (2018). Polymer solar cells: miscibility–function relations in organic solar cells: significance of optimal miscibility in relation to percolation. Adv. Energy Mater..

[21] Lee H, Park C, Sin DH, Park JH, Cho K (2018). Recent advances in morphology optimization for organic photovoltaics. Adv. Mater..

[22] Cheng HW, Zhao Y, Yang Y (2022). Toward high-performance semitransparent organic photovoltaics with narrow-bandgap donors and non-fullerene acceptors. Adv. Energy Mater..

[23] Xu T (2021). 15.8% efficiency binary all-small-molecule organic solar cells enabled by a selenophene substituted sematic liquid crystalline donor. Energy Environ. Sci..

[24] Menke SM, Ran NA, Bazan GC, Friend RH (2018). Understanding energy loss in organic solar cells: toward a new efficiency regime. Joule.

[25] Min J (2016). Side-chain engineering for enhancing the properties of small molecule solar cells: a trade-off beyond efficiency. Adv. Energy Mater..

[26] Gasperini A, Jeanbourquin XA, Rahmanudin A, Yu X, Sivula K (2015). Enhancing the thermal stability of solution-processed small-molecule semiconductor thin films using a flexible linker approach. Adv. Mater..

[27] Griffini G (2011). Long-term thermal stability of high-efficiency polymer solar cells based on photocrosslinkable donor-acceptor conjugated polymers. Adv. Mater..

[28] Kim T, Choi J, Kim HJ, Lee W, Kim BJ (2017). Comparative study of thermal stability, morphology, and performance of all-polymer, fullerene–polymer, and ternary blend solar cells based on the same polymer donor. Macromolecules.

[29] Song J (2020). An optimized fibril network morphology enables high-efficiency and ambient-stable polymer solar cells. Adv. Sci..

[30] Hu H (2020). The role of demixing and crystallization kinetics on the stability of non-fullerene organic solar cells. Adv. Mater..

[31] Lee J-W (2021). Donor–acceptor alternating copolymer compatibilizers for thermally stable, mechanically robust, and high-performance organic solar cells. ACS Nano.

[32] Liu Q (2017). Circumventing UV light induced nanomorphology disorder to achieve long lifetime ptb7-th:pcbm based solar cells. Adv. Energy Mater..

[33] Heumueller T (2015). Disorder-induced open-circuit voltage losses in organic solar cells during photoinduced burn-in. Adv. Energy Mater..

[34] Brabec CJ (2010). Polymer–fullerene bulk-heterojunction solar cells. Adv. Mater..

[35] Wang T (2012). Correlating structure with function in thermally annealed pcdtbt:pc70bm photovoltaic blends. Adv. Funct. Mater..

[36] Duan L, Uddin A (2020). Progress in stability of organic solar cells. Adv. Sci..

[37] Zhang Z (2019). Efficient and thermally stable organic solar cells based on small molecule donor and polymer acceptor. Nat. Commun..

[38] Zhang W (2022). 16.52% Efficiency all-polymer solar cells with high tolerance of the photoactive layer thickness. Adv. Mater..

[39] Zhang ZG, Li Y (2021). Polymerized small-molecule acceptors for high-performance all-polymer solar cells. Angew. Chem. Int. Ed..

[40] Du J (2021). Polymerized small molecular acceptor based all-polymer solar cells with an efficiency of 16.16% via tuning polymer blend morphology by molecular design. Nat. Commun..

[41] Wang G, Melkonyan FS, Facchetti A, Marks TJ (2019). All-polymer solar cells: recent progress, challenges, and prospects. Angew. Chem. Int. Ed..

[42] Liu B (2022). Backbone configuration and electronic property tuning of imide-functionalized ladder-type heteroarenes-based polymer acceptors for efficient all-polymer solar cells. Adv. Funct. Mater..

[43] Lee J-W (2019). Origin of the high donor–acceptor composition tolerance in device performance and mechanical robustness of all-polymer solar cells. Chem. Mater..

[44] Choi J, Kim W, Kim S, Kim T-S, Kim BJ (2019). Influence of acceptor type and polymer molecular weight on the mechanical properties of polymer solar cells. Chem. Mater..

[45] Ma R (2022). Achieving high efficiency and well-kept ductility in ternary all-polymer organic photovoltaic blends thanks to two well miscible donors. Matter.

[46] Wu Y (2021). A conjugated donor-acceptor block copolymer enables over 11% efficiency for single-component polymer solar cells. Joule.

[47] Sun Z (2011). PS-b-P3HT copolymers as P3HT/PCBM interfacial compatibilizers for high efficiency photovoltaics. Adv. Mater..

[48] Zhang Q, Cirpan A, Russell TP, Emrick T (2009). Donor−acceptor poly (thiophene-block-perylene diimide) copolymers: synthesis and solar cell fabrication. Macromolecules.

[49] Gu Z, Kanto T, Tsuchiya K, Shimomura T, Ogino K (2011). Annealing effect on performance and morphology of photovoltaic devices based on poly (3-hexylthiophene)-b-poly (ethylene oxide). J. Polym. Sci., Part A: Polym. Chem..

[50] Park SH (2020). Significantly improved morphology and efficiency of nonhalogenated solvent-processed solar cells derived from a conjugated donor–acceptor block copolymer. Adv. Sci..

[51] Park CG (2019). Facile one-pot polymerization of a fully conjugated donor–acceptor block copolymer and its application in efficient single component polymer solar cells. J. Mater. Chem. A.

[52] Sun H (2021). Regioregular narrow-bandgap n-type polymers with high electron mobility enabling highly efficient all-polymer solar cells. Adv. Mater..

[53] Zhang Y, Tajima K, Hirota K, Hashimoto K (2008). Synthesis of all-conjugated diblock copolymers by quasi-living polymerization and observation of their microphase separation. J. Am. Chem. Soc..

[54] Xia X (2022). Multidimensional control of repeating unit/sequence/topology for one-step synthesis of block polymers from monomer mixtures. J. Am. Chem. Soc..

[55] Li S (2021). Narrow-bandgap single-component polymer solar cells with approaching 9% efficiency. Adv. Mater..

[56] Wu Y (2022). Non-fullerene acceptor doped block copolymer for efficient and stable organic solar cells. ACS Energy Lett..

[57] Wu Z (2016). n-Type water/alcohol-soluble naphthalene diimide-based conjugated polymers for high-performance polymer solar cells. J. Am. Chem. Soc..

[58] Rivnay J, Mannsfeld SC, Miller CE, Salleo A, Toney MF (2012). Quantitative determination of organic semiconductor microstructure from the molecular to device scale. Chem. Rev..

[59] Boudouris BW (2011). Real-time observation of poly (3-alkylthiophene) crystallization and correlation with transient optoelectronic properties. Macromolecules.

[60] Schilinsky P, Waldauf C, Brabec CJ (2002). Recombination and loss analysis in polythiophene based bulk heterojunction photodetectors. Appl. Phys. Lett..

[61] Li B (2022). Over 16% efficiency all-polymer solar cells by sequential deposition. Sci. China Chem..

[62] Gu KL (2018). Nanoscale domain imaging of all-polymer organic solar cells by photo-induced force microscopy. ACS Nano.

[63] Zhu L (2022). Single-junction organic solar cells with over 19% efficiency enabled by a refined double-fibril network morphology. Nat. Mater..

[64] Zhang C (2019). Comprehensive investigation and analysis of bulk-heterojunction microstructure of high-performance PCE11: PCBM solar cells. ACS Appl. Mater. Interfaces.

[65] Du X (2019). Efficient polymer solar cells based on non-fullerene acceptors with potential device lifetime approaching 10 years. Joule.

[66] Li N (2017). Abnormal strong burn-in degradation of highly efficient polymer solar cells caused by spinodal donor-acceptor demixing. Nat. Commun..

[67] Zhang K-N (2021). Exploring the mechanisms of exciton diffusion improvement in ternary polymer solar cells: From ultrafast to ultraslow temporal scale. Nano Energy.

[68] Li D, Zhang X, Liu D, Wang T (2020). Aggregation of non-fullerene acceptors in organic solar cells. J. Mater. Chem. A.

[69] Weng K (2020). Optimized active layer morphology toward efficient and polymer batch insensitive organic solar cells. Nat. Commun..

[70] Wang Z (2021). The coupling and competition of crystallization and phase separation, correlating thermodynamics and kinetics in OPV morphology and performances. Nat. Commun..

[71] Yang C (2020). Molecular design of a non-fullerene acceptor enables a P3HT-based organic solar cell with 9.46% efficiency. Energy Environ. Sci..

[72] He Y (2022). Unraveling the charge-carrier dynamics from the femtosecond to the microsecond time scale in double-cable polymer-based single-component organic solar cells. Adv. Energy Mater..

[73] Feng G (2019). Thermal-driven phase separation of double-cable polymers enables efficient single-component organic solar cells. Joule.

[74] Guo C (2013). Conjugated block copolymer photovoltaics with near 3% efficiency through microphase separation. Nano Lett..

