# Supplementary material for: Efficient and stable organic solar cells enabled by multicomponent photoactive layer based on one-pot polymerization
Source: Nat Commun. 2023 Feb 21;14:967. doi: 10.1038/s41467-023-36413-3 (PMC9944902; doi:10.1038/s41467-023-36413-3)
Supplement: Supplementary file 1 — Supplementary Information [file 41467_2023_36413_MOESM1_ESM.pdf]

Supplementary Information

For

Efficient and stable organic solar cells enabled by multicomponent photoactive layer based on one-pot polymerization

Liu et al.

1. Supplementary Methods.

Monomer Synthesis of BTI-Tin. Fresh LDA (2.5 mL, 2.1 mmol) was added dropwise at -78 ^{o}C to the mixture of bithiophene imide (BTI, 460 mg, 1.00 mmol) in 10 mL THF. The reaction mixture was stirred at -78 ^{o}C for 1 h, (n-Bu)$_3$SnCl (0.7 mL, 2.20 mmol) in THF was added into the reaction dropwise at -78 ^{o}C over 1 h, then the reaction mixture was warmed slowly to room temperature and stirred at this temperature overnight. The reaction mixture was quenched with 40 mL H_2O and extracted with $CHCl_3$ (3 × 50 mL), the combined organic layer was then washed with saturated $NaHCO_3$ aq (20 mL) and H_2O (40 mL). The organic layer was dried over anhydrous Na_2SO_4. After the removal of the solvent under vacuum, the residue was purified over flash column chromatography on silica gel with petroleum ether as the eluent to afford monomer BTI-Tin (622 mg, 60%) as a colorless oil. ^{1}H NMR (400 MHz, $CDCl_3$) δ (ppm): 7.74 (s, 2H), 4.23 (d, J= 8.0 Hz, 2H), 1.96-1.90 (m, 1H), 1.62-1.54 (m, 12H) 1.39-1.07 (m, 48H), 0.91 (t, J= 4.0 Hz 18H), 0.88-0.82 (m, 6H). ^{1}H NMR data is consistent with the previous results[1].

Monomer Synthesis of Y5-Br. Compound a (200 mg, 0.14 mmol, Supplementary Fig. 1), IC-Br (159.6 mg, 0.6 mmol), pyridine (1 mL) and chloroform (25 mL) were added to a flask under N_2. The reaction was stirred at 65 °C for 12 h. After cooled to room temperature, the reaction mixture was poured into methanol and filtered. The residue was purified with column chromatography on silica gel using dichloromethane: petroleum ether (1:1, v:v) as the eluent to give a dark blue solid as the product **Y5-Br** (209 mg, 80% yield). ^{1}H NMR (400 MHz, $CDCl_3$) δ (ppm): 9.15 (s,2H), 8.54 (d, J = 8.0 Hz, 2H), 8.03 (d, J = 2.0 Hz, 2H), 7.84 (dd, J = 8.4, 1.9 Hz, 2H), 4.78 (d, J = 7.6 Hz, 4H), 3.20 (t, J = 7.7 Hz, 4H), 2.14 (s, 2H), 1.91-1.82 (m, 4H), 1.54-1.48 (m, 4H),1.40-0.76 (m, 108H). ^{1}H NMR data is consistent with the previous results[2].

Polymerization of L15. To a 20 mL dry microwave tube equipped with a stirring bar was added distannylated monomer BTI-Tin (0.1 mmol), dibrominated monomer Y5-Br (187 mg, 0.1 mmol), $Pd_2(dba)_3$ (1.37 mg, 0.0015 mmol), P(*o*-tolyl)$_3$ (3.65 mg, 0.012 mmol), and toluene (8 mL). The tube was purged with argon and sealed under argon flow. The reaction tube was then loaded into a microwave reactor and heated to 110 °C and stirred at this temperature for 3 h. 0.2 mL 2-bromothiophene was then added and the reaction mixture was stirred at 110 ºC for another 0.5h. Finally, 2-(tributylstannyl)thiophene was added and stirred at 140 ºC for another 0.5 h. After cooled to room temperature, the reaction solution was dripped into 80 mL methanol containing 2 mL HCl (12 N) under vigorous stirring and stirred for 1 h. The precipitate was dried in vacuo and further purified by silica gel column chromatography by using $CHCl_3$ as eluent. The polymer was then precipitated in methanol (60 mL) and dried under a vacuum for 12 h before use (184.5 mg, yield: 85%). ^1H NMR (400 MHZ, $C_2D_2Cl_4$, 80 °C) δ: 8.36-8.33 (m, 1H), 8.22-8.05 (m, 2H), 7.62-7.57 (m, 2H), 7.43-7.29 (m, 2H), 7.24-7.13 (m, 2H), 6.80-6.73 (m, 1H), 6.57-6.46 (m, 1H), 4.29-4.22 (m, 4H), 3.76-3.50 (m, 6H), 2.00-0.30 (m, 187H). ^1H NMR data is consistent with the previous results[3].

Synthesis of PM6 block. In a 50 mL dry flask, $Pd(PPh_3)_4$ (10 mg) was added to a solution of d_1 (93.8 mg, 0.1 mmol) and a_1 (76.4 mg, 0.1 mmol) in 5 mL degassed toluene under argon flow. The PM6 block can be obtained via stirred vigorously at 110°C for 2 hours without further purification. M_n = 6.9 kDa, M_w = 12.9 kDa, and PDI = 1.85. It can be seen form Supplementary Fig. 7 that the ratio of BDT and BDD unit in the PM6 block is about 1:1, indicating the successful coupling reaction. Besides, Br end groups and trimethyl tin are concurrently coupled in the PM6 block, which is of great importance for synthesizing the final block copolymer.

Polymerization of PM6-*b*-PY-IT and S9. In an inert atmosphere, *d*1 (Supplementary Fig. 2, 477.79 mg, 0.5 mmol), *a*1 (383.36 mg, 0.5 mmol) and $Pd(PPh_3)_4$ (4 mg) were first dissolved in anhydrous toluene (5 mL) in a 50 mL reaction flask. The reaction

mixture was heated to 110 °C and stirred at this temperature for 2h. The mixture of *a*2 (937.21 mg, 0.5 mmol) and *d*2 (331.12 mg, 0.5 mmol) in 15 mL toluene was injected into the above reaction flask, and the reaction mixture was stirred at 110 °C for another 24 h. The resulting solution was cooled and added to methanol dropwise (200 mL). The precipitates were subjected to different post-processing method. For the PM6-*b*-PY-IT block polymer, the precipitated solid was filtered by a thimble and subjected to the sequential Soxhlet extraction with methanol, hexane and dichloromethane as the solvents to remove low molecular weight fractions. The residue was extracted with chloroform as the final solvent and the chloroform solution was concentrated to 20 mL and precipitated into 100mL methanol. The precipitate was dried in vacuo to yield the product polymer. The obtained polymer was dissolved in chloroform and further purified through silica gel column with chloroform. The collected chloroform solution was concentrated and precipitated in methanol to obtain PM6-*b*-PY-IT (1.07 g, yield: 71%). M_n = 16.4 kDa, M_w = 32.8 kDa, and PDI = 2.00. Regarding the multicomponent material S9, a simple flash column chromatography with chloroform as eluent to remove residual small amounts of monomers and catalyst. The fuscous solid was dried in vacuo to yield the product S9 (1.16 g, yield: 77%). M_n = 16.3 KDa, M_w = 43.8 kDa, PDI = 2.69. The molar ratio of the PM6 and PY-IT units in the materials was determined by Energy-dispersive X-ray spectroscopy (EDX) and X-ray photoelectron spectroscopy (XPS). The F and N elements only exist in the PM6 and PY-IT, respectively, we thus use the content of fluorine and nitrogen to acquire the repeated elemental analyses. According to the XPS spectra of PM6-*b*-PY-IT and S9 in Supplementary Fig. 12, the ratios of F and N concentration are 1:2.47 and 1:2.70, indicating that the ratios of PM6 block and PY-IT block in the final systems are around 1:0.84 and 1:0.92. Besides, the EDX results show that the molar ratios of the PM6 and PY-IT blocks were determined about 1:0.82 and 1:0.89 in PM6-*b*-PY-IT and S9 systems (Supplementary Fig. 13). The block lengths are calculated to be 6 and 5 (52% and 48%) for PM6 and PY-IT blocks in the corresponding and PM6-*b*-PY-IT block copolymers[4].

Polymerization of PM6-*b*-L15 and S11. In an inert atmosphere, *d*1 (477.79 mg, 0.5 mmol), *a*1 (383.36 mg, 0.5 mmol) and $Pd(PPh_3)_4$ (4 mg) were first dissolved in anhydrous toluene (5 mL) in a 50 mL reaction flask. The reaction was heated to 110 °C and stirred at this temperature for 2h. The mixture of *a*2 (937.21 mg, 0.5 mmol) and *a*3 (548.80 mg, 0.5 mmol) in 15 mL toluene was then injected into the reaction flask, and the reaction mixture was stirred at 110 °C for another 24 h. The resulting solution was cooled and added to methanol dropwise (200 mL). The precipitates were subjected to different post-processing method. For the PM6-*b*-L15 block polymer, this block copolymer is purified as the method for PM6-*b*-PYIT described above. PM6-*b*-L15 (1.14 g, yield: 67%), M_n = 18.6 kDa, M_w = 57.2 kDa, and PDI = 3.07. Regarding S11 multicomponent material, it is purified as the method for S9 described above. The fuscous solid was dried in vacuo to yield the product S11 (1.24 g, yield: 73%). M_n = 18.6 kDa, M_w = 50.4 kDa and PDI = 2.71.

As shown in Supplementary Fig. 8, the characteristic signals for PM6 and L15 repeating units appear simultaneously in the ^{1}H NMR spectra of PM6-*b*-L15 and S11, such as 2.01-2.51 ppm for the aliphatic peaks in PM6, 3.50-4.94 ppm for the aliphatic peaks and 7.62-8.91 ppm for aromatic peaks in L15 block. The ^{1}H NMR results demonstrate the presence of both PM6 and L15 blocks in their corresponding block copolymers. According to the XPS spectra of PM6-*b*-L15 and S11in Supplementary Fig. 12, the ratios of F and N concentration for the PM6-*b*-L15 and S11 systems are 1:2.72 and 1:3.03, demonstrating that the ratios of PM6 block and L15 block for the corresponding materials are ~1:0.82 and 1:0.91, respectively. In addition, the EDX results show that the molar ratios of the PM6 and L15 repeating units are around 1:0.84 and 1:0.91 for the target PM6-*b*-L15 and S11 products (Supplementary Fig. 12 and Supplementary Table 2). It can be found that the elemental analysis results of EDX conform well with those of the XPS data. The block lengths are calculated to be 6 and 5 (52% and 48%) for PM6 and L15 blocks in the corresponding and PM6-*b*-L15 block copolymers[S4].

The composition analysis of the multicomponent system S11. To clarify the composition of the multicomponent system S11, the resultant material was post-processed *via* the sequential simple flash column chromatography and Soxhlet extraction. As illustrated in Supplementary Fig. 11, the photoactive material of S11 was first developed *via* the post-treatment of the simple flash column chromatography to remove the residual palladium catalysts and unreacted monomers. We denoted the total mass content of the S11 multicomponents as M_s. It can be found that the fluorine (F) element only exists in the PM6 segment of PM6-*b*-L15 and in the PM6 block, and the nitrogen (N) element is in the L15 segment of PM6-*b*-L15 and in the L15 block. According to the XPS spectra of the S11 material in Supplementary Fig. 12, the ratio of F and N contents for the S11 system is 1:3.03, demonstrating that the mass content percentages of L15 (block and segment) and PM6 (block and segment) for the corresponding material is 47.64% and 52.36% (1:0.91).

Next, the S11 photoactive material was further post-treated *via* the Soxhlet extraction to remove the PM6 and L15 blocks with low molecular weight[5,6]. Therefore, the purified block copolymer of PM6-*b*-L15 was obtained, and there was an 8.0 % total mass loss (92.0 % M_s) compared to the S11 system. As shown in Supplementary Table 4, the measured mass content of N element in PM6-*b*-L15 is 2.67%, while the theoretical mass content of N in PM6-*b*-L15 molecular formula is 3.72%. Besides, the XPS results show that the molar ratio of the PM6 and L15 repeating units is around 1:0.84 for the target PM6-*b*-L15 products. Based on these data, the mass content percentages of L15 segment and PM6 segment of the PM6-*b*-L15 polymer in S11 system were calculated to be 42.35% and 50.42%. Therefore, the mass contents of the dissociative (or residual) PM6 block and L15 block in S11 were determined to be 1.94% and 5.29% (Supplementary Table 5). That is, our materials are multicomponent mixtures including PM6 blocks (~2%), L15 blocks (~5%) and PM6-*b*-L15 block polymers (~93%).

Batch-to-batch variation of S11. To probe into the effects of molecular weight change of donor and acceptor block on polymer properties and device performances, various batches of S11 are synthesized accordingly. The PM6 blocks with different weight-average molecular weights (M_{Ws}) can be obtained by controlling the polymerization reaction time of d_1 and a_1 (t_1). Then a_2 and a_3 were added to extend the conjugated main chain and polymerize for a certain time (t_2), forming

multicomponent systems with various conjugated lengths of PM6 and L15 blocks (Supplementary Fig. 16).

(1) Fixing the reaction time of PM6 blocks at 2 h, and changing the polymerization time of L15 blocks.

Through fixing t_1 polymerization time at 2 h and regulating t_2 reaction periods, three batches of the block copolymers for S11-A1 (t_2 = 24 h), S11-A2 (t_2 = 12 h), and S11-A3 (t_2 = 8 h) were successfully achieved. The molecular weight and PDI of the target S11 materials measured by GPC were summarized in Supplementary Fig. 17 and Supplementary Table 6. The M_Ws of S11-A1, S11-A2, and S11-A3 were measured as 50.4, 30.7, and 25.3 kDa with PDI values of 2.71, 2.16, and 2.24, respectively. To study the photovoltaic performances of these polymers, OSCs were fabricated with a device structure of ITO/PEDOT:PSS/S11/PNDIT-F3N/Ag. As summarized in Supplementary Fig. 18 and Supplementary Table 7, the corresponding power conversion efficiencies (PCEs) were found to be 11.76%, 11.53% and 11.06%, respectively. The weak dependence of PCE on M_w clearly indicates good repeatability of this polymerization approach when fixing the reaction time of donor blocks.

The PCE values of the S11-based OSCs show good batch repeatability when fixing the reaction time of donor blocks, demonstrating the good reproducibility of their photovoltaic properties.

(2) Changing the reaction time of PM6 blocks, and fixing the total polymerization periods at 26 h.

Three batches of the copolymers for S11-D1 (t_1 =1 h), S11-D2 (t_1 =2 h), and S11-D3 (t_1 =3 h) were also obtained when fixing the total polymerization periods at 26 h (t_1+t_2 =26 h) and adjusting the reaction time of PM6 blocks. As shwn in Supplementary Fig. 19 and Supplementary Table 8, the M_Ws of S11-D1, S11-D2, and S11-D3 were measured as 82.8, 50.4, and 42.3 kDa with PDI values of 3.09, 2.71, and 2.63, respectively. From the analysis of the GPC results, compared to S11-D2 and S11-D3, the shortest PM6 block of S11-D1 showed the highest M_w. This phenomenon is mainly caused by the weakened chain propagation reactivity when the length of the PM6 block becomes longer[7]. As illustrated in Supplementary Fig. 20 and Supplementary Table 9, the PCEs of the OSCs increased from 6.27% to 11.72% as the length of PM6 block increased. While the polymerization period reaches 3 h, the S11-D3-based devices exihibit a much lower PCE of 8.65 %.

Moreover, the UV-vis absorption spectra of the S11 polymers in chloroform solutions

and thin films were recorded (Supplementary Fig. 21). With the increase of t_1, the peak value of PM6 gradually sharpened, and the intensity ratio of PM6 (donor block) to L15 (acceptor block) (I_d/I_a) gradually increased. The I_d/I_a less than 1 was observed in the absorption spectra of S11-D1 and S11-D2, while the intensity of the PM6 block in S11-D3 is much greater than the L15 block. Different from the PM6 donor block which formed push-pull effect after polymerizing d_1 and a_1, the monomer a_2, featuring the A-D-A-D-A structure itself, has a strong push-pull effect and thus narrow bandgap with high extinction coefficient, which demonstrates a weaker dependence of absorption intensity on block length in L15 block than those of PM6 block.

Characterization Analysis. NMR spectra of monomers and polymers were recorded on Bruker Ascend 400 MHz spectrometer. High-resolution mass spectrometry was obtained on Thermo ScientificTM Q-Exactive. The molecular weight of the polymers was measured using a high-temperature gel permeation chromatography (GPC, Agilent PL-GPC220) at 150 °C with 1,2,4-trichlorobenzene as the eluent and polystyrenes as the standards. UV-vis absorption spectra of polymer solution and films were recorded on a Shimadzu UV-3600 UV-VIS-NIR spectrophotometer. The cyclic voltammetry was used to determine the polymer energy levels in thin-film under argon atmosphere using a CHI760 Voltammetry Workstation with a saturated solution of 0.1 M tetra(n-butyl) ammonium hexafluorophosphate (Bu_4NPF_6) in acetonitrile (CH_3CN) as the supporting electrolyte. The energy levels were calculated using the following formulas of (1) and (2):

$$E_{LOMO} =- e(E_{red}^{onset} + 4.80)eV \qquad (1)$$

$$E_{HOMO} =- e(E_{ox}^{onset} + 4.80)eV \qquad (2)$$

The grazing incidence wide angle x-ray scattering (GIXS) analysis was conducted in the Pohang Accelerator Laboratory (beamline 3C, Republic of Korea), with incidence angle between 0.12 - 0.14°. L_c values of the materials were calculated using Scherrer equation (3):

$$L_c = \frac{2\pi K}{\Delta_q} \qquad (3)$$

(K (shape factor) = 0.9 and Δ_q = full width half maximum (FWHM) of the scatterings)

OFET Device Fabrication and Characterization. Top-gate/bottom-contact (TG/BC) organic thin-film transistors were fabricated to investigate electron transport characteristics of the S9 and S11 polymers. Source/drain electrodes of 3 nm Cr/30 nm Au were patterned on borosilicate glass by photolithography, with a channel length of 10, 20, 50, or 100 μm and a channel width of 5 mm. Next, the polymers are spin-coated (3000 rpm, 60 s) from their chlorobenzene solution (5 mg mL^{-1}). The polymer films were thermally annealed at various temperatures for 10 min followed by a cooling-down quenching process. Then, ~280 nm amorphous fluoropolymer CYTOP was coated on top and annealed at 100 °C for 20 min. Finally, 50 nm Al was evaporated on top as the gate electrode to complete the device fabrication. The OTFT characterization was performed inside an N_2-filled glove box using a Keithley S4200 semiconductor analyzer. To extract saturation mobility, the standard equation (4) was used:

$$I_{sd} = \mu_{sat} C_i \left(\frac{W}{L}\right) \left(V_g - V_{th}\right) V_{sd} \qquad (4)$$

where I_{sd} is the source/drain current, μ_{sat} is the saturation mobility, W is the channel width, L is the channel length, V_g is the gate voltage, V_{th} is the threshold voltage, and V_{sd} is the source/drain voltage (V_{sd} = 80 V for saturation regime). It should be noted that the charge transport in OFET devices is in the in-plane (or lateral) direction. Different from OFETs, it was considered that the charge transport was along the out-of-plane (or vertical) direction in OPVs. This nature determined the annealing temperature difference of the optimum OFET and OSC investigation[8,9].

SCLC Mobility Measurement. Hole and electron mobilities were measured using the space-charge-limited-current (SCLC) method. The structure of ITO/PEDOT:PSS/active layer/MoO_3/Ag was used for hole-only devices and the structure of ITO/ZnO/active layer/Ca/Al was used for electron-only devices, respectively. The SCLC mobilities were calculated by the MOTT-Gurney equation (5):

$$J = \frac{9}{8}\varepsilon_{\gamma}\varepsilon_{0}\mu\frac{V^{2}}{L^{3}} \qquad (5)$$

Where J is the current density, ε_r is the relative dielectric constant of active layer material, usually 2- 4 for organic/polymer semiconductors, herein we use a relative dielectric constant of 3 for polymer. ε_0 is the permittivity of space, μ is the mobility of hole or electron and d is the active layer thickness, V is the internal voltage in the device, and $V = V_{appl} - V_{bi}$, where V_{appl} is the voltage applied to the device, and V_{bi} is the built-in voltage resulting from the relative work function difference between the two electrodes (in the hole-only and the electron-only devices, the V_{bi} values can be neglected).

Characterization Analysis of Film Properties. The AFM, TEM, and GIWAX characterizations were employed to compare the morphology difference for PM6:L15 and S11 films. As shown in Supplementary Fig. 31 a and e, S11 film shows a more textured surface compared to that of PM6:L15 active layer, leading to a large domain size and coarser surface roughness (RMS) (1.81 nm *versus* 0.93 nm). Moreover, more defined fibrillar nanostructures for S11 active layer can be observed than that for PM6:L15 system in AFM phase images (Supplementary Fig. 31 b and f). The TEM photograph of S11 exhibits more distinctive and uniform microphase-separated textures than the binary blend (Supplementary Fig. 31 c and g), which may be advantageous for the maintenance of morphology stability. In both of the two films, the π-π (010) diffraction peaks appear in the out-of-plane direction, demonstrating a preference of face-on orientation (Supplementary Fig. 31 d and h). The calculated crystal coherence length (L_c) values are summarized in Supplementary Table 16. The PM6:L15 film showed higher L_c values in both (100) peak along the in-plane direction (21.2 nm) and (010) peak along the out-of-plane direction (4.27 nm) than the S11 system (21.6 nm and 3.85 nm), leading to improved charge transporting ability in OSCs. The GIWAX results provide a better explanation for the relatively low *J-V* performance of S11-based OSCs.

Moreover, to better understand the hole transfer kinetics in the systems, the transient absorption spectroscopy (TAS) was correspondingly performed. The TA spectra of PM6:L15 and S11 films after photoexcitation at 700 nm with 7.5 μJ cm^{-2} $pulse^{-1}$ at selected delay times are exhibited in Supplementary Fig. 32 a and b, and the hole transfer dynamics of the two systems at 598 nm were extracted in Supplementary Fig. 32 c. The PM6:L15 blend achieved higher transfer and yielded longer transfer time, demonstrating greatly increased amounts of hole transfer in the PM6:L15 blend film with prolonged exciton diffusion distances[10,11]. The hole transfer TA results are in accordance with the photovoltaic parameters of PM6:L15 and S11 systems.

2. Supplementary Figures

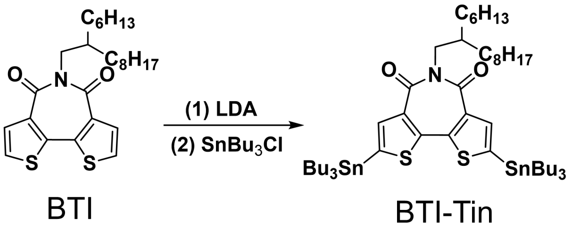

Supplementary Fig. 1 Synthetic route to BTI-Tin monomer.

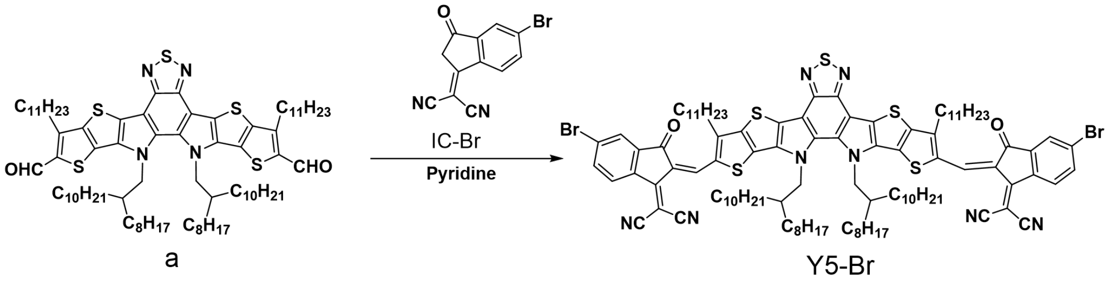

Supplementary Fig. 2 Synthetic route to Y5-Br monomer.

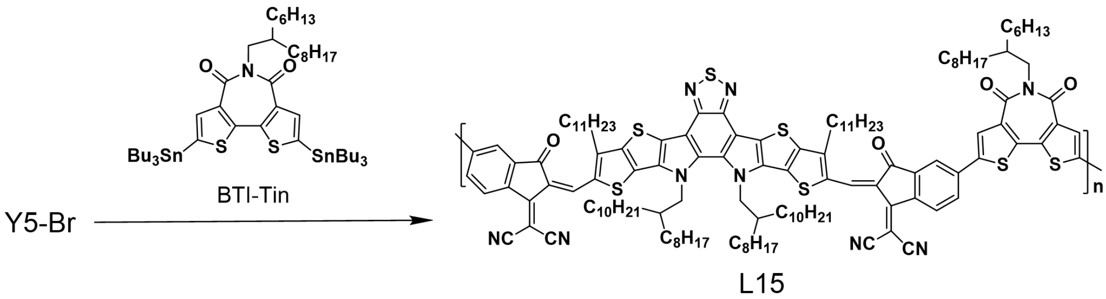

Supplementary Fig. 3 Synthetic route to polymer acceptors L15.

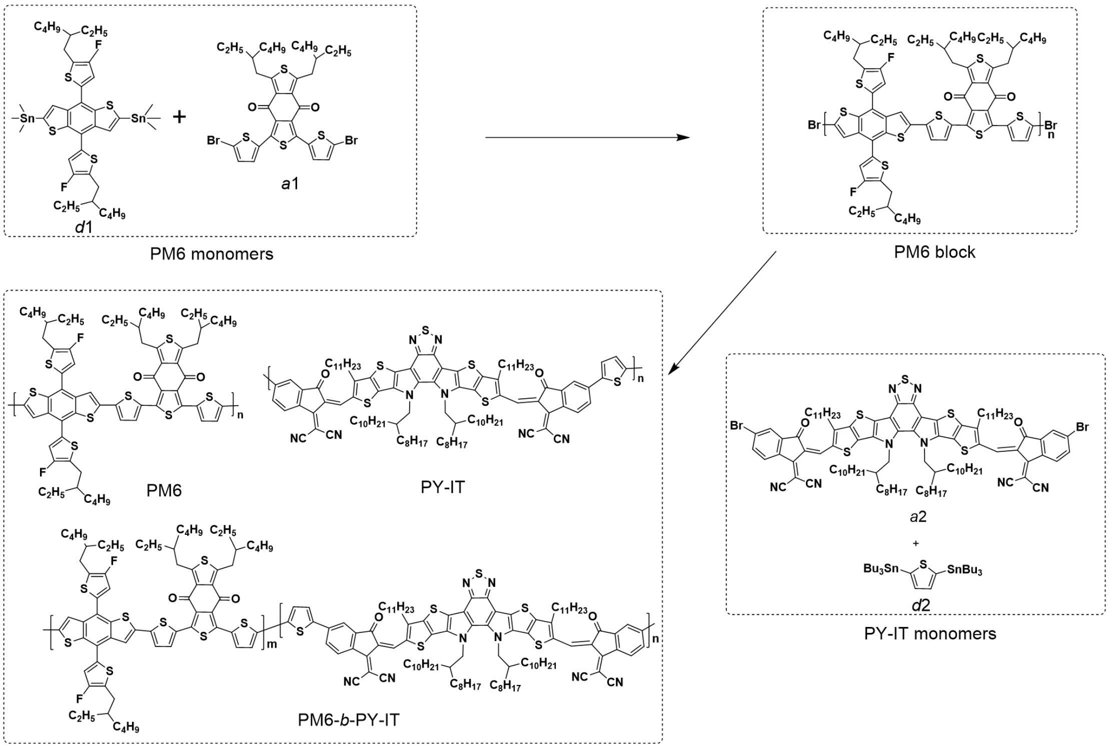

S9

Supplementary Fig. 4 Synthetic route to multicomponent photoactive layer of S9.

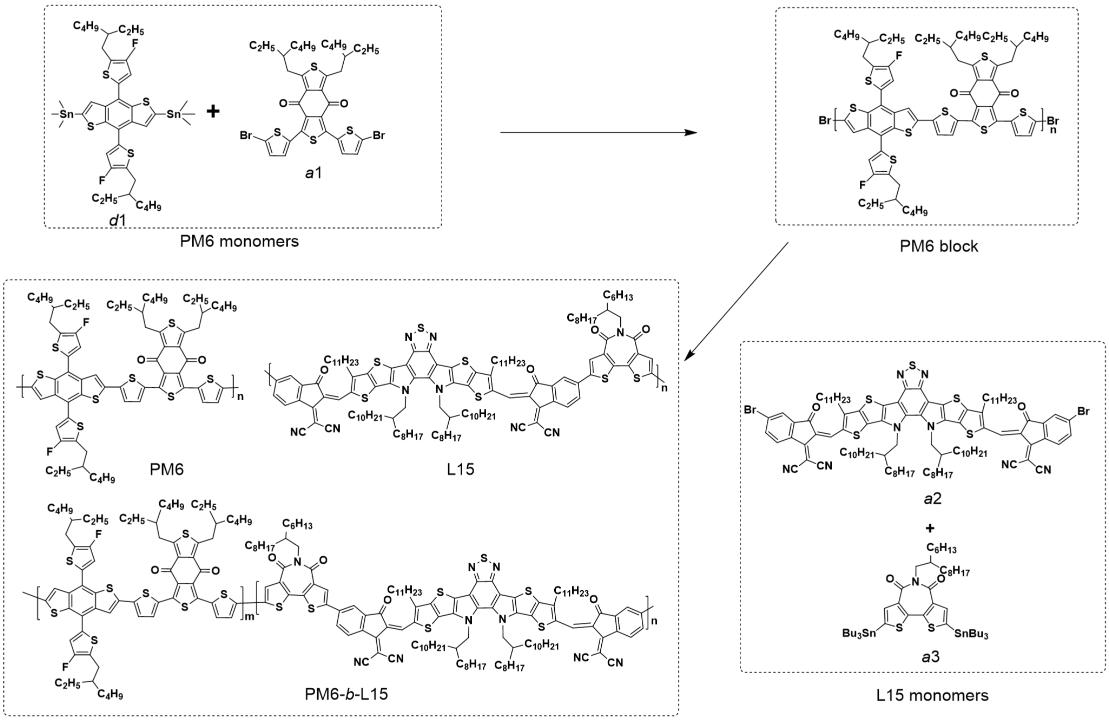

S11

Supplementary Fig. 5 Synthetic route to multicomponent photoactive layer of S11.

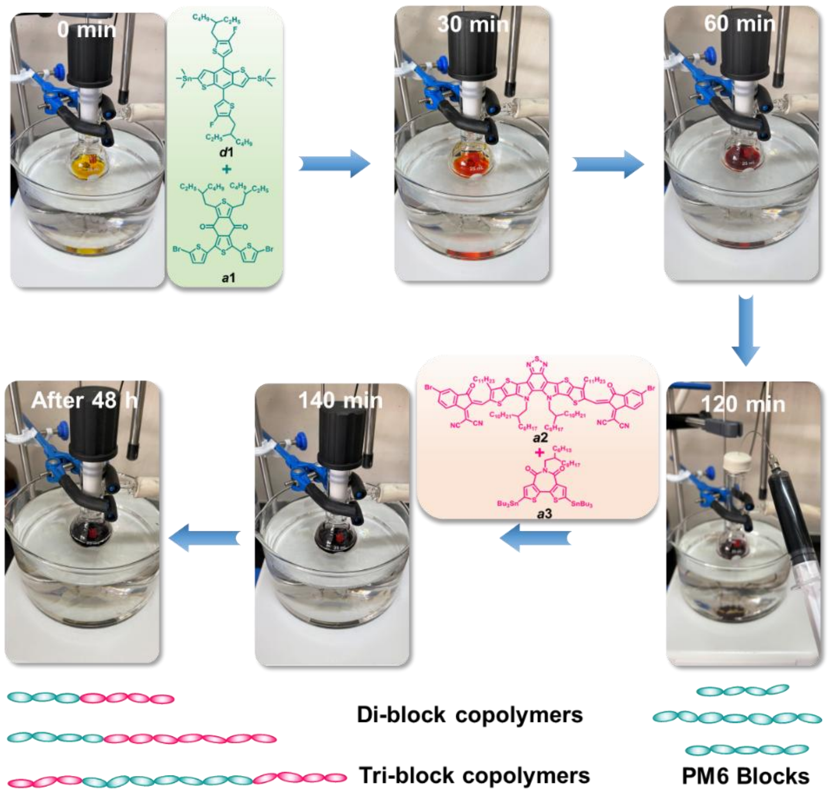

Supplementary Fig. 6 Photographic images showing the progress of one-pot polymerization.

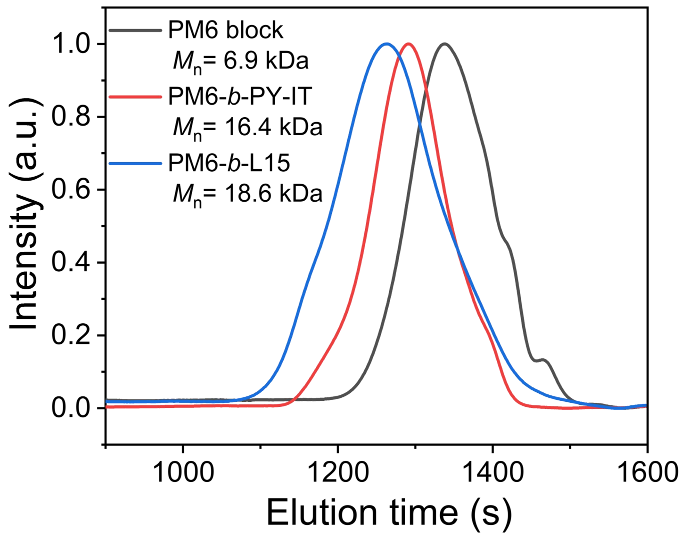

Supplementary Fig. 7 GPC chromatograms of the PM6 block and target block copolymers of PM6-*b*-PY-IT and PM6-*b*-L15.

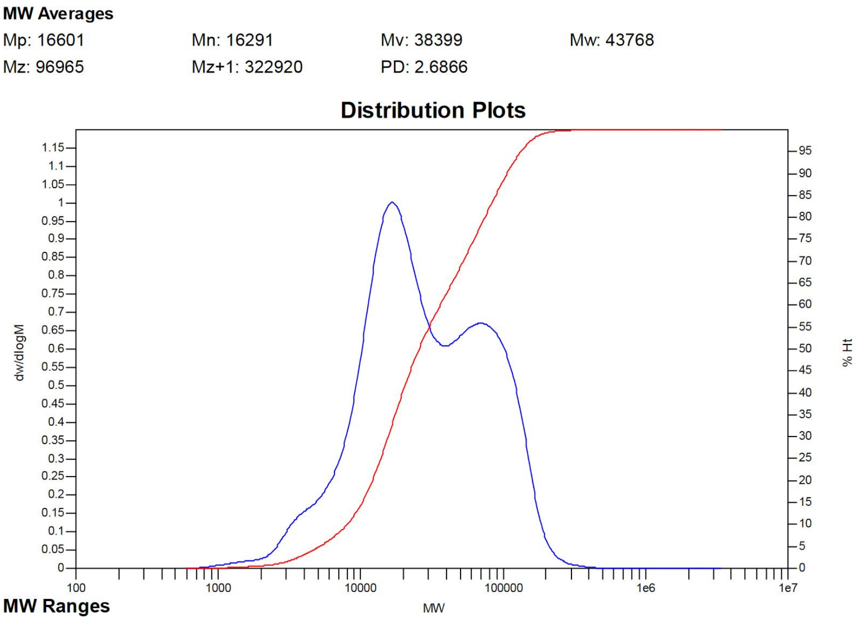

Supplementary Fig. 8 GPC curve of multicomponent photoactive layer S9.

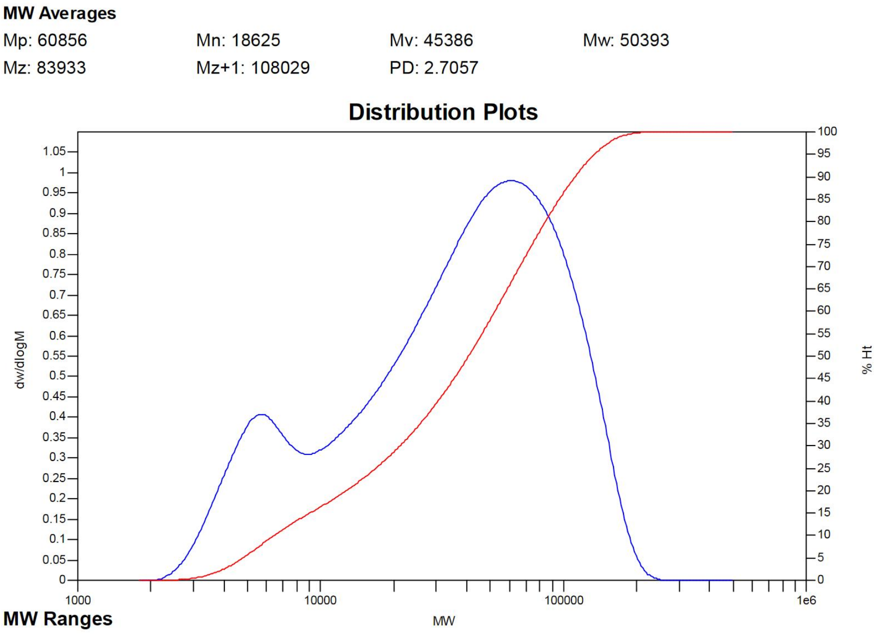

Supplementary Fig. 9 GPC curve of multicomponent photoactive layer S11.

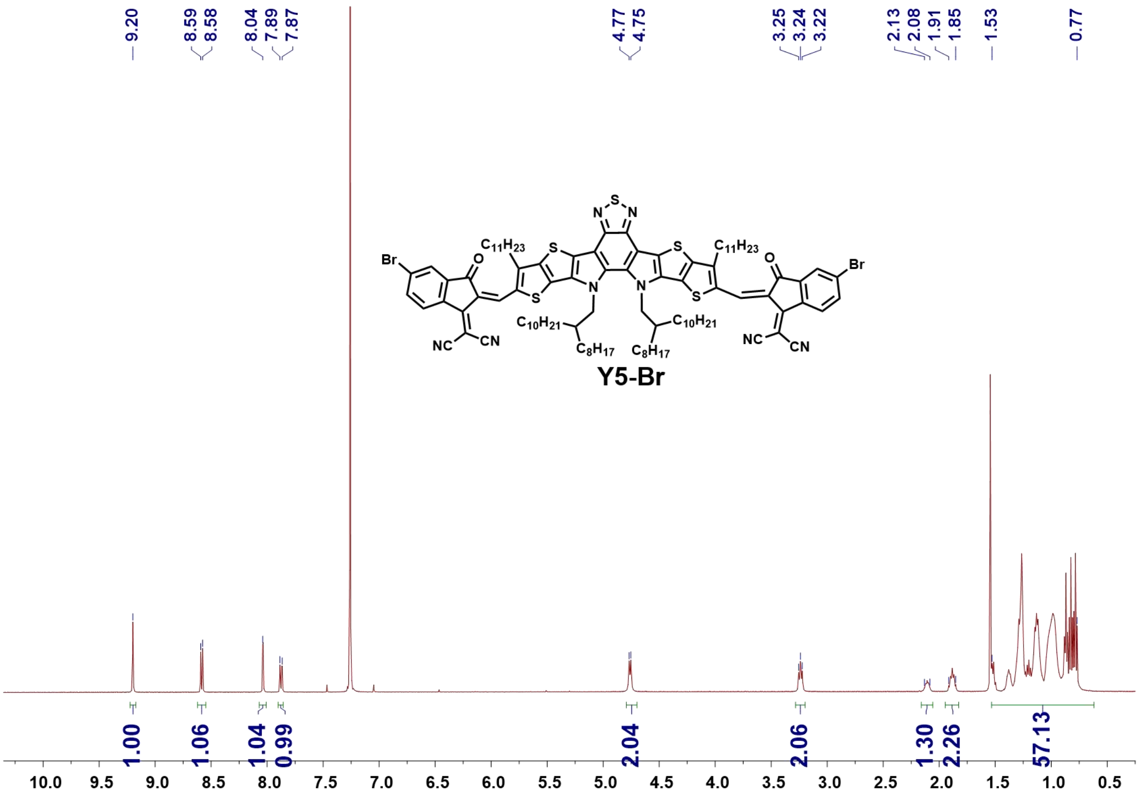

Supplementary Fig. 10 ^{1}H NMR spectrum of Y5-Br.

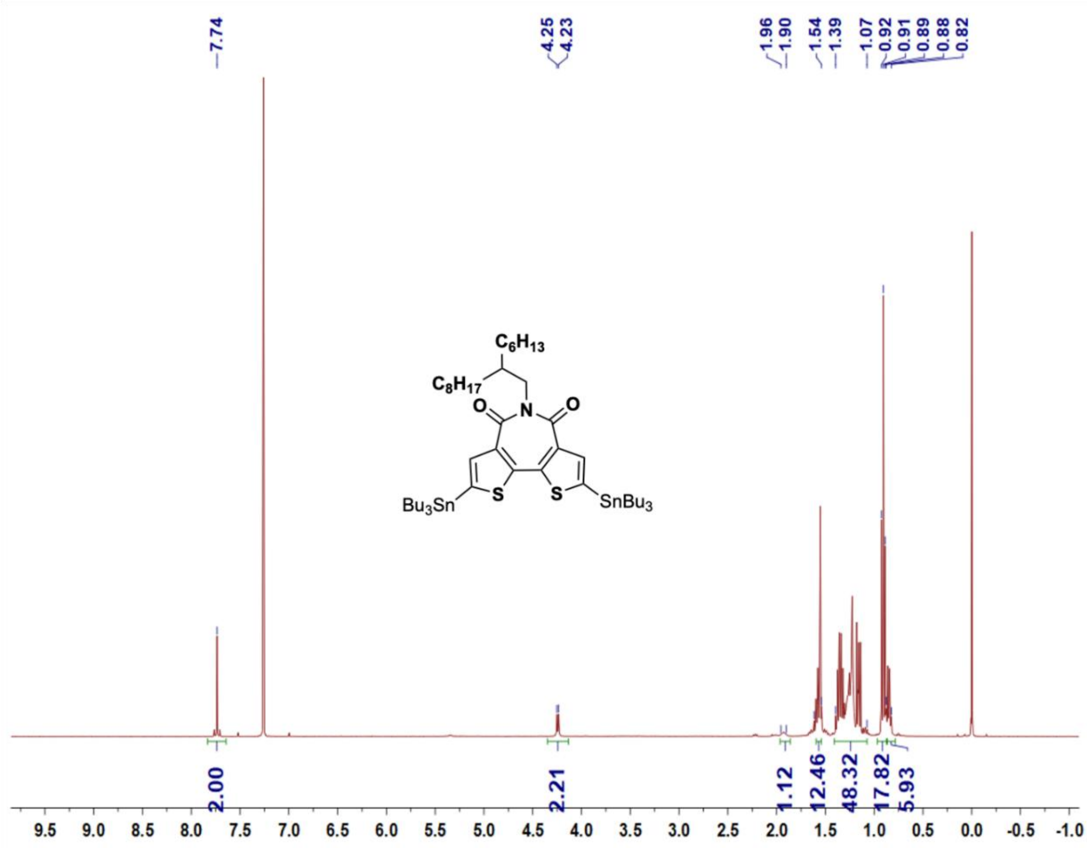

Supplementary Fig. 11 ^{1}H NMR spectrum of BTI-tin.

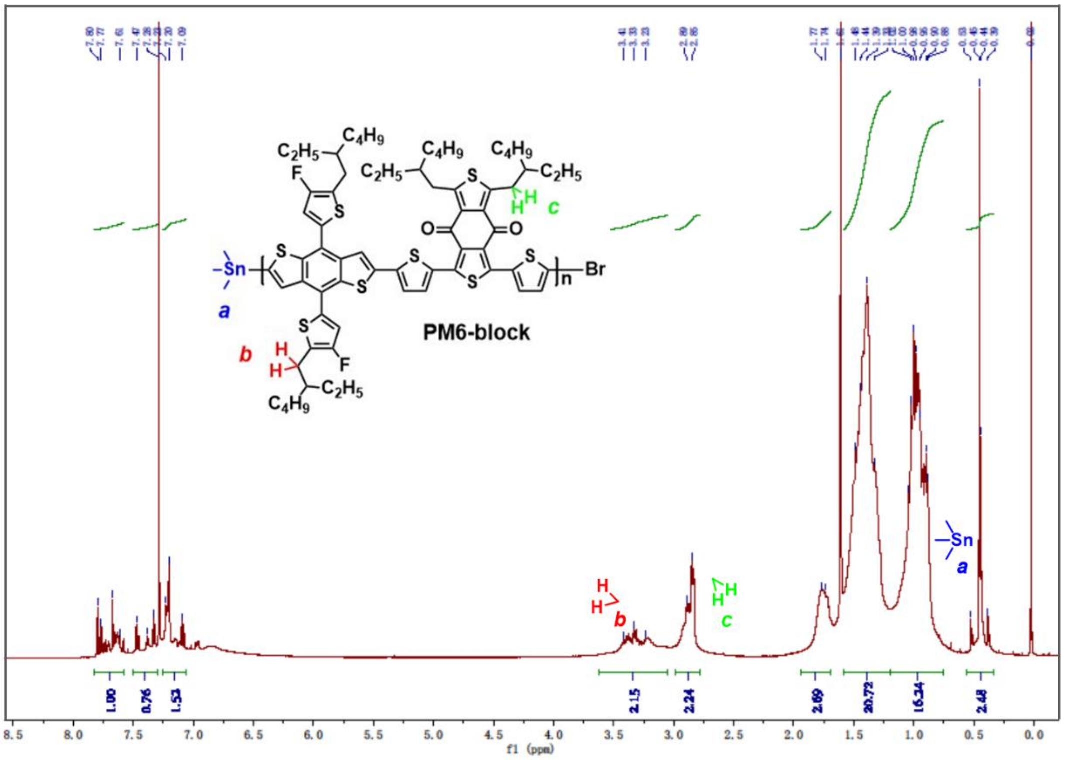

Supplementary Fig. 12 ^{1}H NMR spectra of PM6 block (Solvent: $C_2D_2Cl_4$).

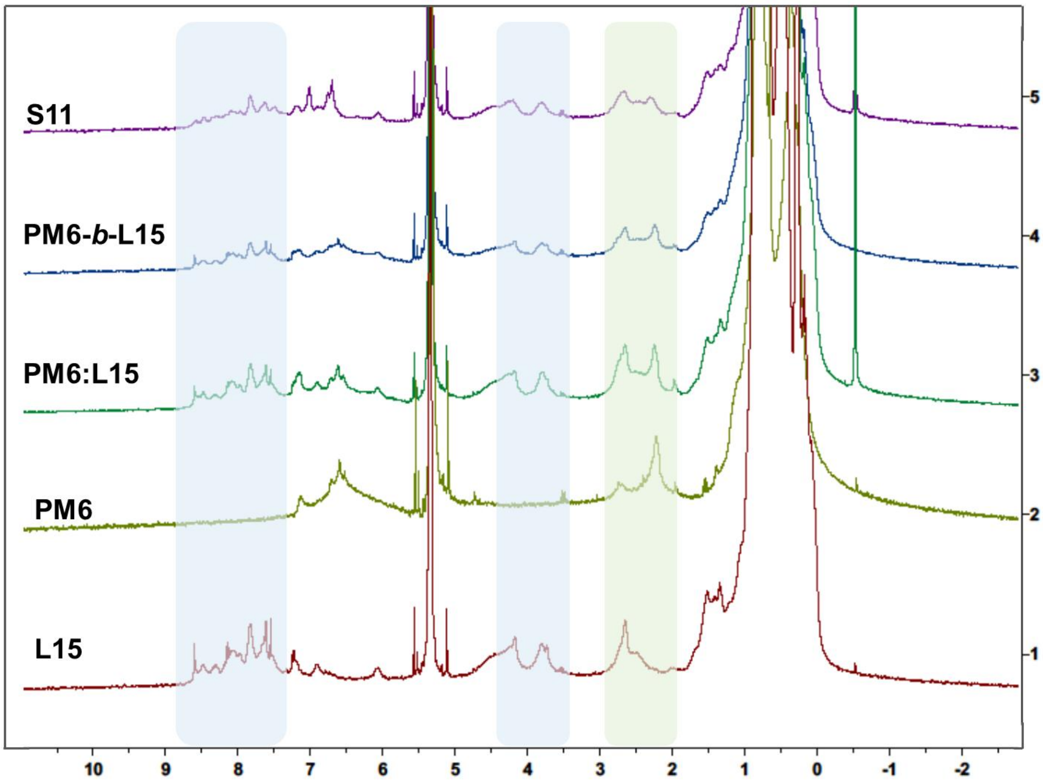

Supplementary Fig. 13 ^{1}H NMR spectra of PM6, L15, PM6:L15 and PM6-*b*-L15 (Solvent: $C_2D_2Cl_4$).

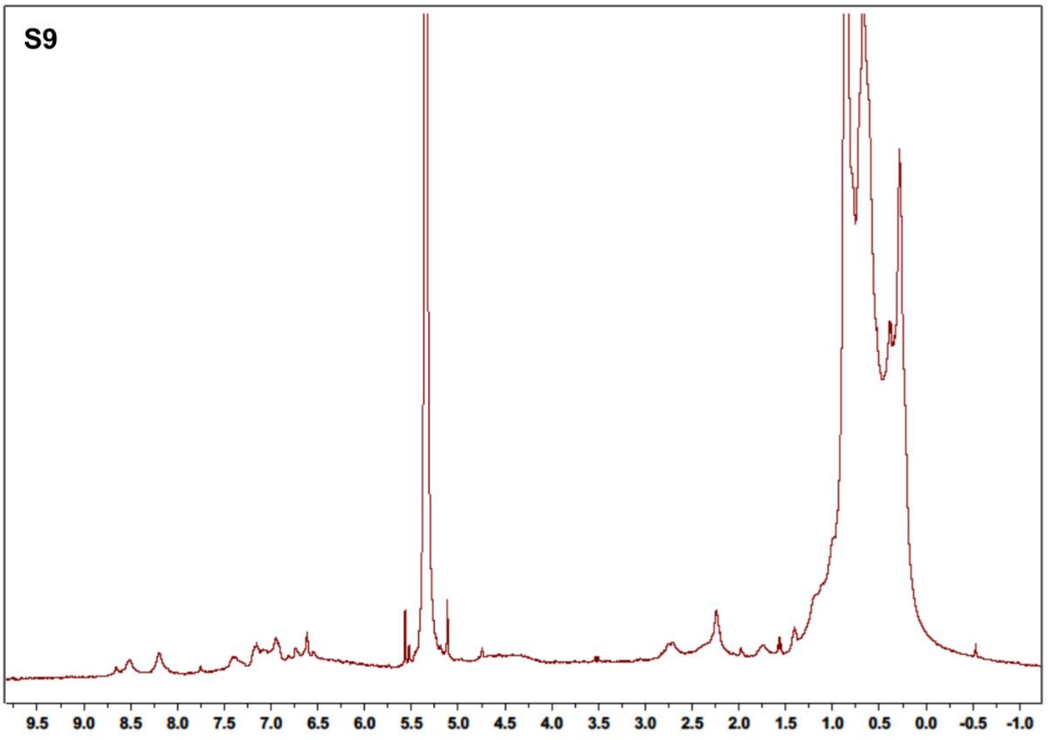

Supplementary Fig. 14 ^{1}H NMR spectra of S9 (Solvent: $C_2D_2Cl_4$).

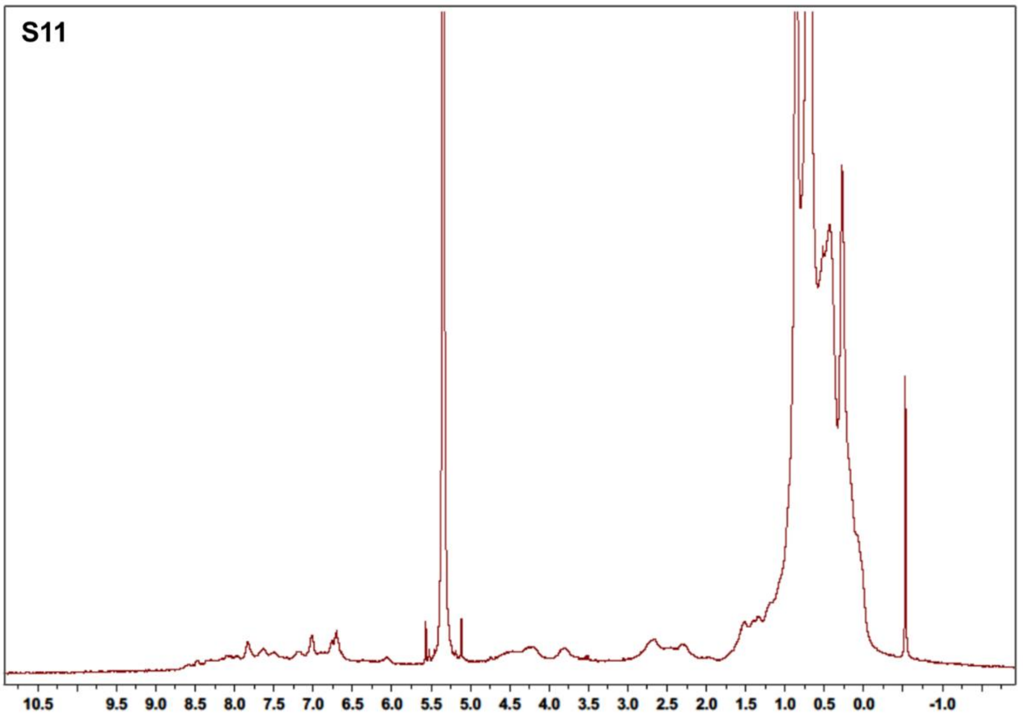

Supplementary Fig. 15 ^{1}H NMR spectra of S11 (Solvent: $C_2D_2Cl_4$).

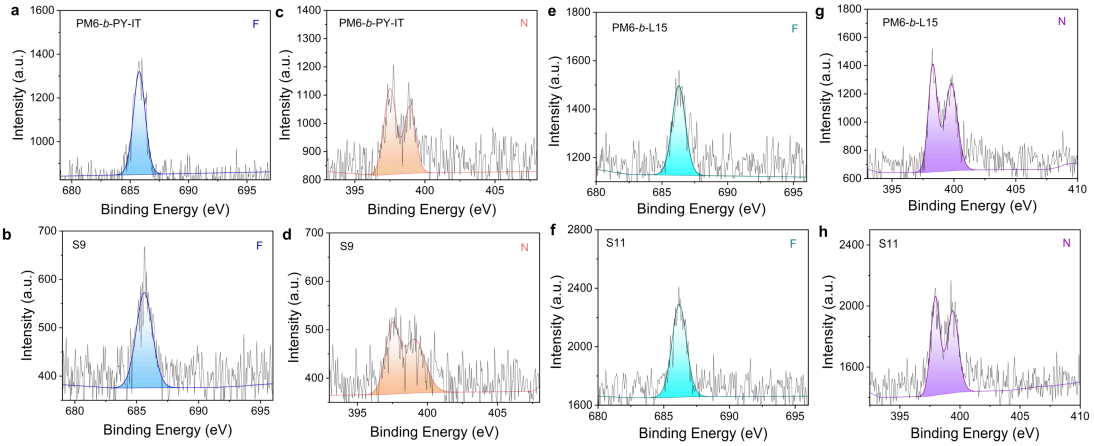

Supplementary Fig. 16 XPS spectra. **a** F *1s* spectrum and **c** N *1s* spectrum of PM6-*b*-PYIT block polymer. **b** F *1s* spectrum and **d** N *1s* spectrum of multicomponent system S9. **e** F *1s* spectrum and **g** N *1s* spectrum of PM6-*b*-L15 block polymer. **f** F *1s* spectrum and **h** N *1s* spectrum of multicomponent system S11.

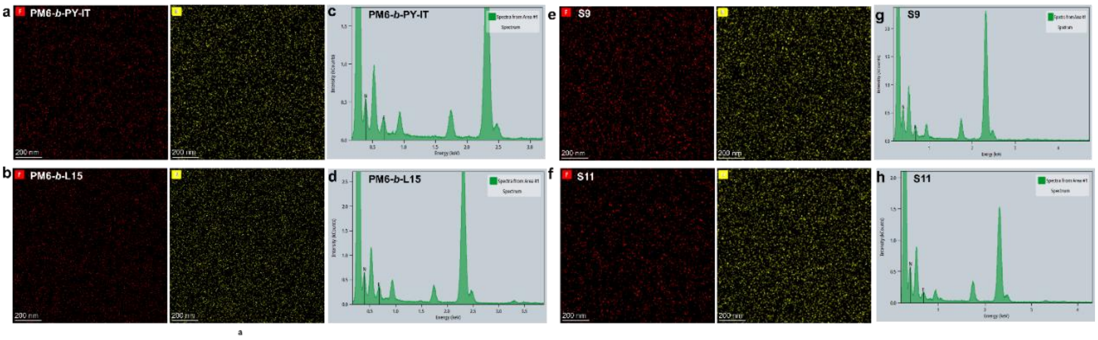

Supplementary Fig. 17 Energy dispersive X-ray (EDX) mapping analysis of F and N elements for **a** PM6-*b*-PYIT, **b** PM6-*b*-L15, **e** S9 and **f** S11photoactive materials. EDX analysis of **c** PM6-*b*-PYIT, **d** PM6-*b*-L15, **g** S9 and **h** S11 thin film. The scale bar is 200 nm.

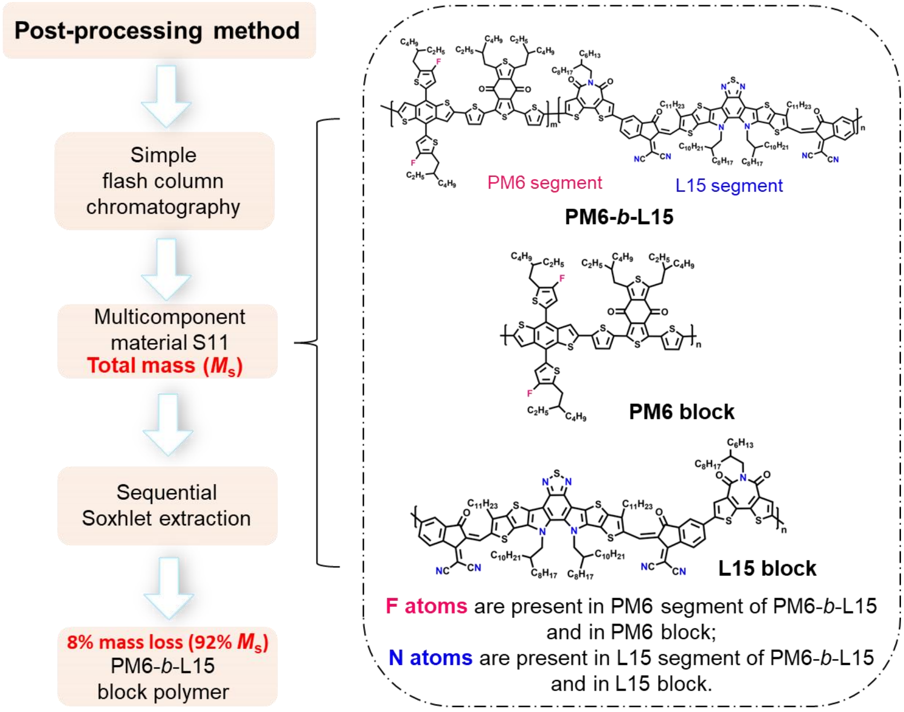

Supplementary Fig. 18 The schematic diagram of post-polymerization processing for the multicomponent system and block copolymer synthesized via one-pot polymerization.

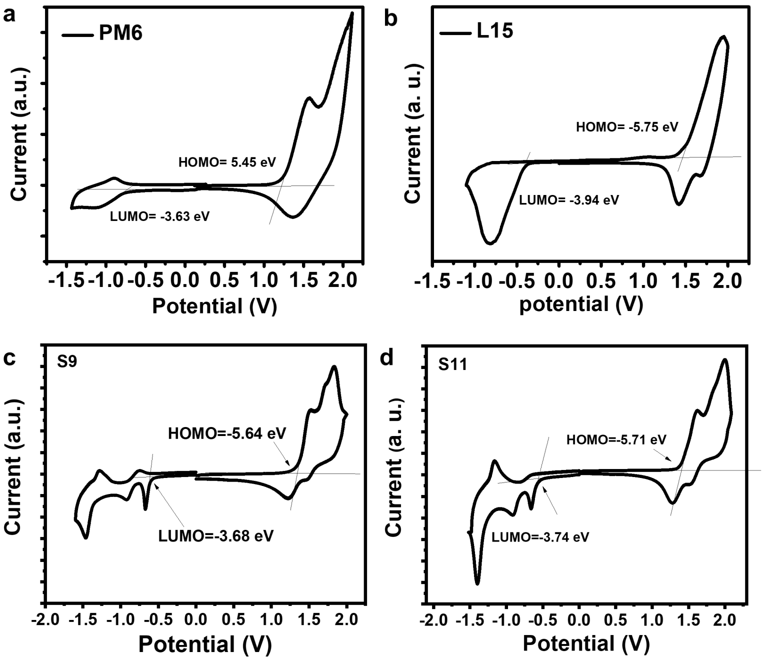

Supplementary Fig. 19 The cyclic voltammetry of (a) PM6, (b) L15, (c) S9 and (d) S11.

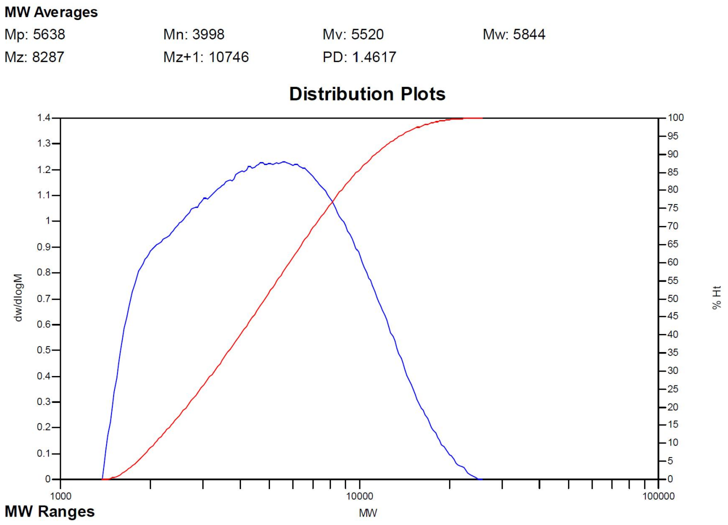

Supplementary Fig. 20 GPC curve of the polymer PNDIT-F3N.

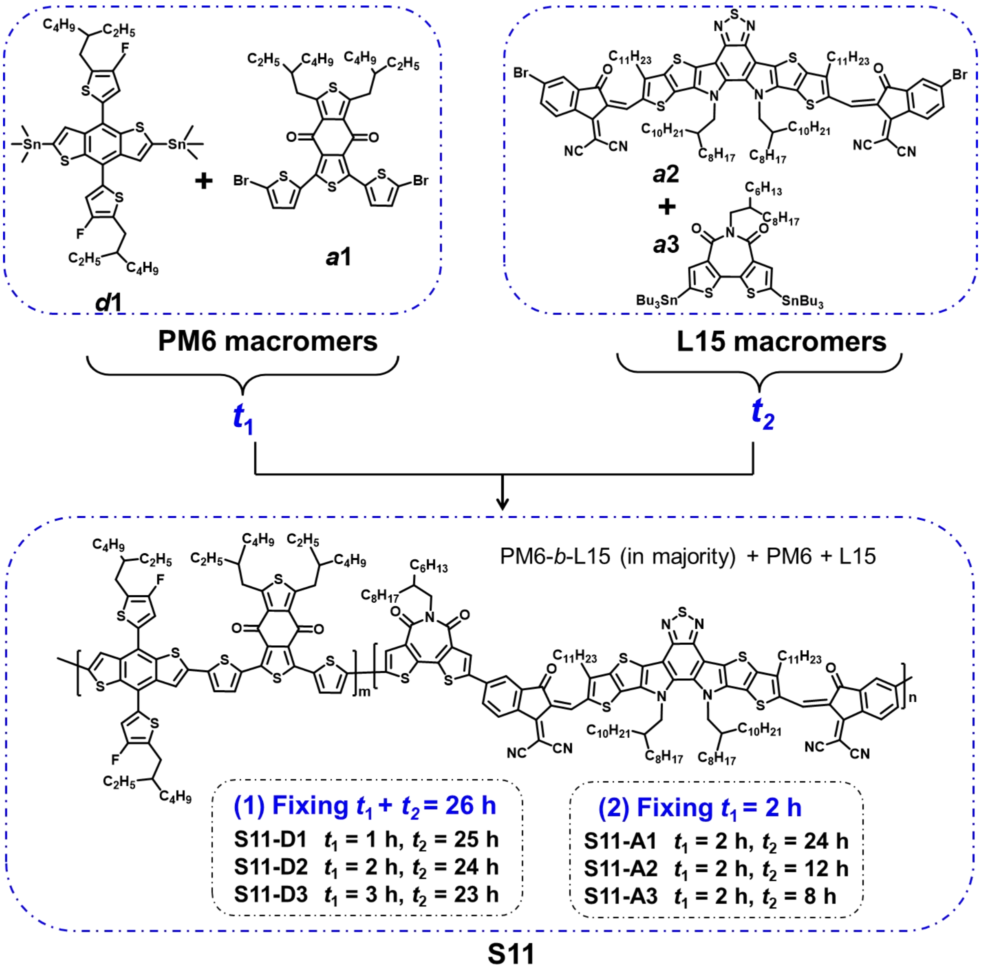

Supplementary Fig. 21 The synthetic routes to the multicomponent system S11.

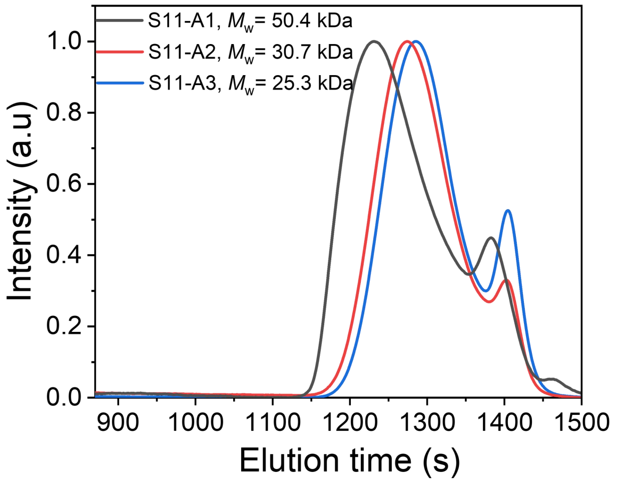

Supplementary Fig. 22 GPC chromatograms of the different batches of S11.

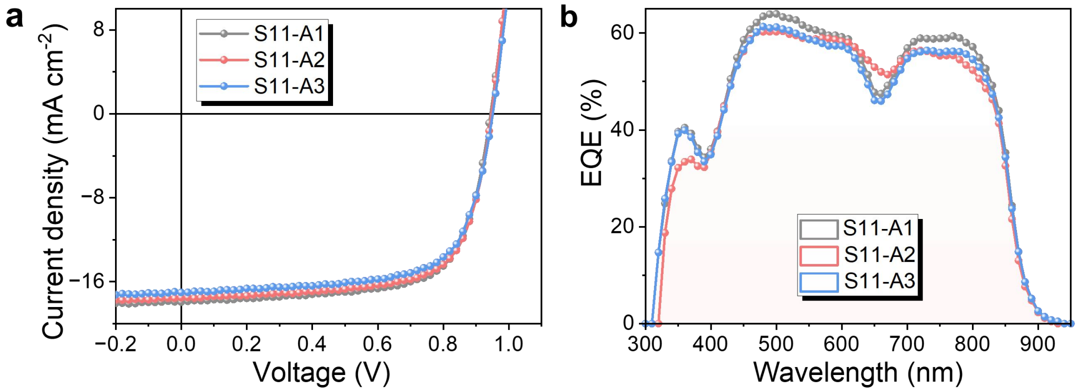

Supplementary Fig. 23 **a** *J–V* characteristics for OSCs based on various batches of S11. **b** The EQE spectra for the corresponding devices.

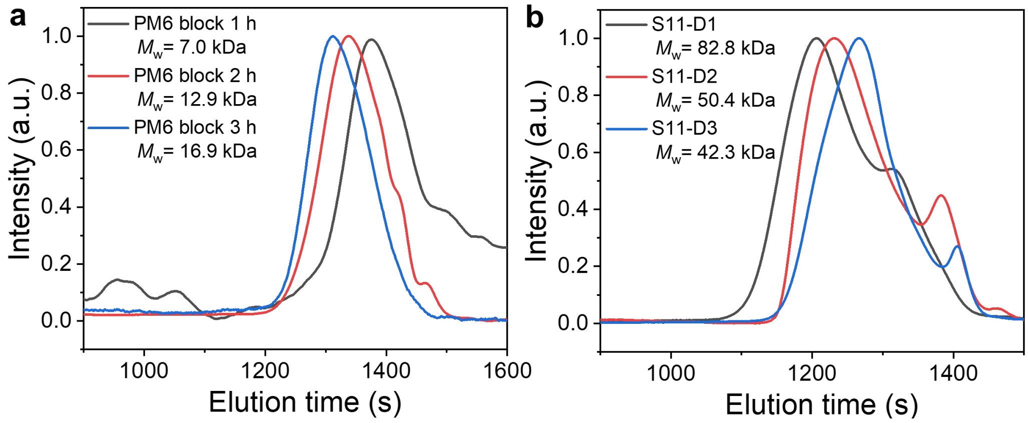

Supplementary Fig. 24 The molecular weights of **a** PM6 blocks and **b** S11 with different polymerization periods.

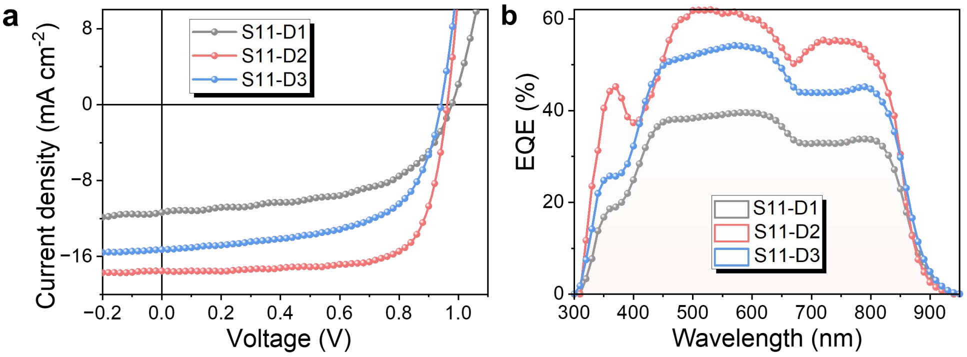

Supplementary Fig. 25 a *J–V* characteristics for OSCs based on different batches of S11. **b** The EQE spectra for the corresponding devices.

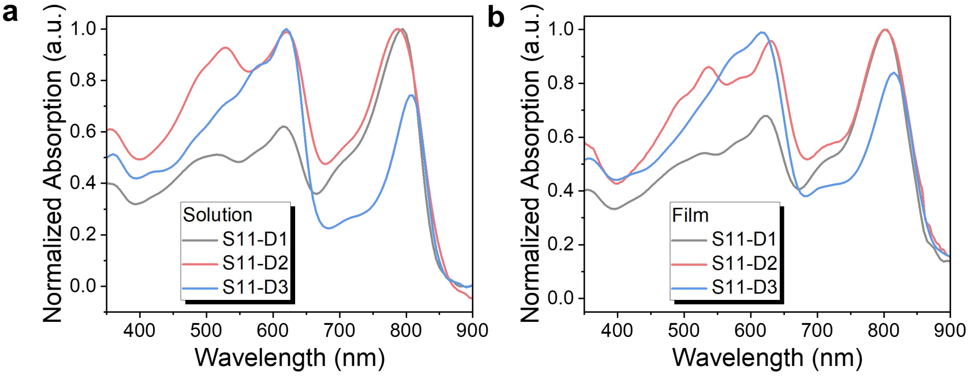

Supplementary Fig. 26 The normalized absorption spectra of different batches of S11 in **a** dilute chloroform solutions and **b** the solid states.

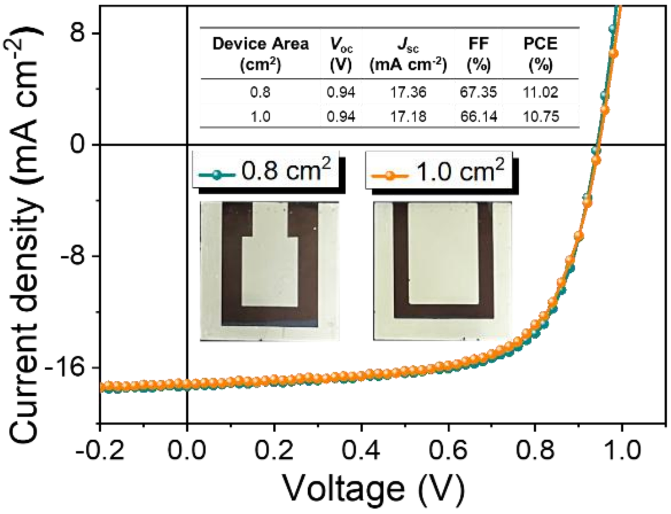

Supplementary Fig. 27 *J-V* curves and performance parameters for large-area OSCs with 0.8 and 1.0 cm^2 active area. The insets are the images for the corresponding devices.

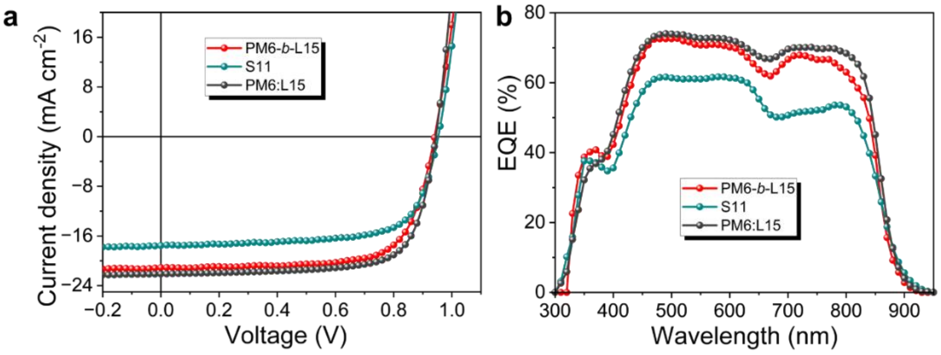

Supplementary Fig. 28 a *J–V* characteristics and **b** EQE spectra for OSCs based on PM6:L15, PM6-b-L15 and S11 photoactive layers.

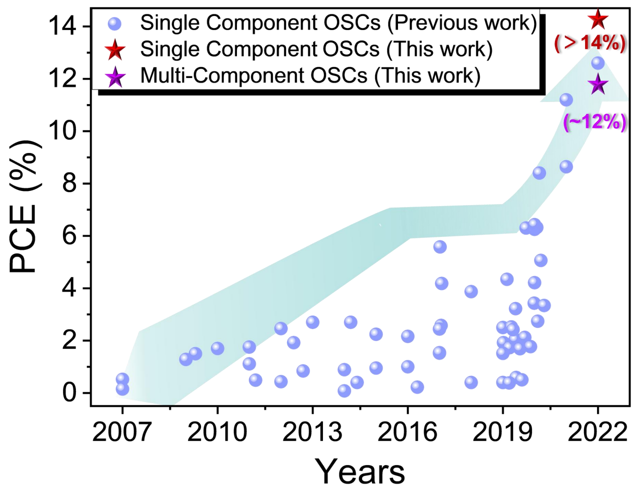

Supplementary Fig. 29 Plots of the PCE development for single-component OSCs and our multicomponent OSCs in this work.

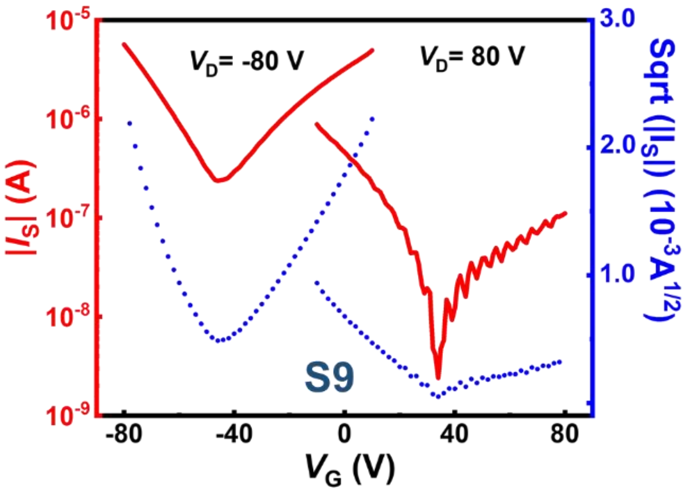

Supplementary Fig. 30 Transfer characteristics of S9-based top-gate/bottom-contact (TGBC) organic field-effect transistors (OFETs).

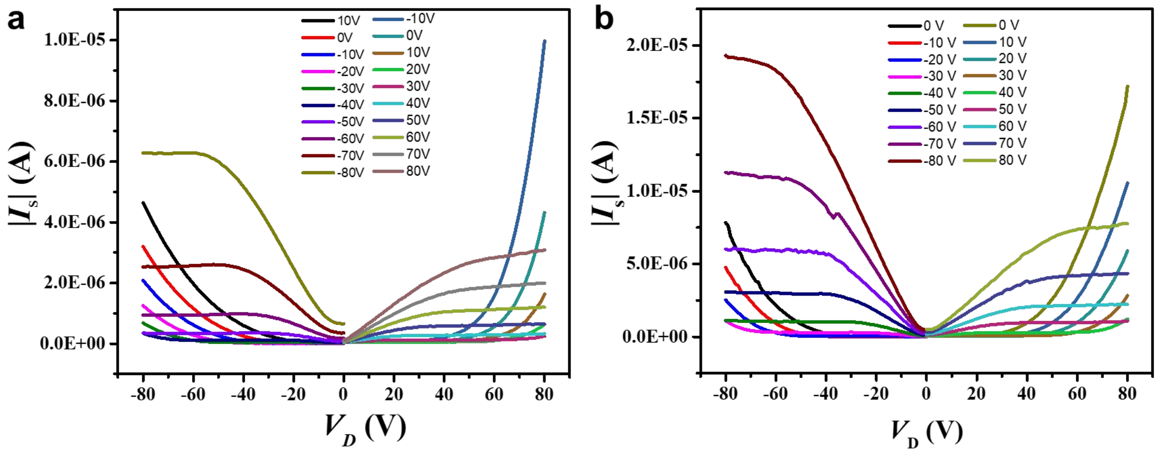

Supplementary Fig. 31 TG/BC OFET output characteristics of **a** S9 and **b** S11.

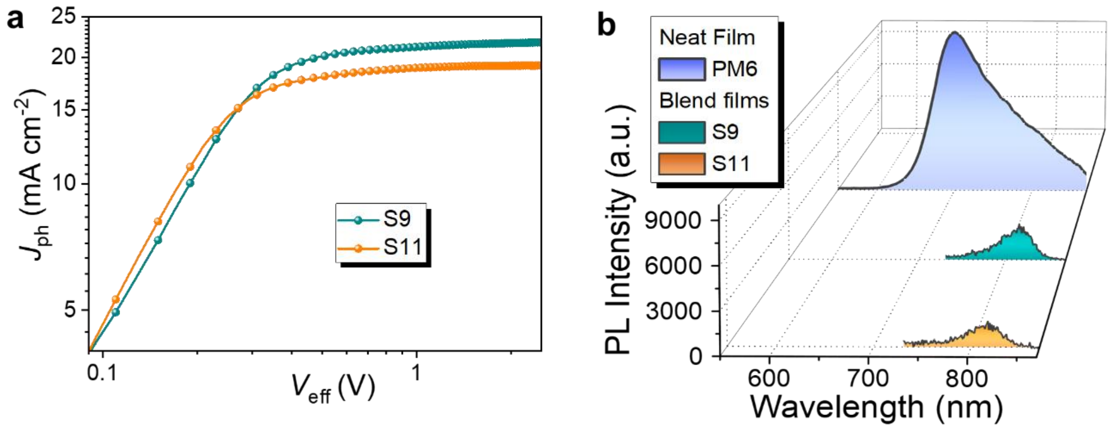

Supplementary Fig. 32 a J_{ph}-V_{eff} curves of OSCs. **b** Photoluminescent (PL) spectra of PM6 neat film, S9 and S11 photoactive layers excited at 450 nm.

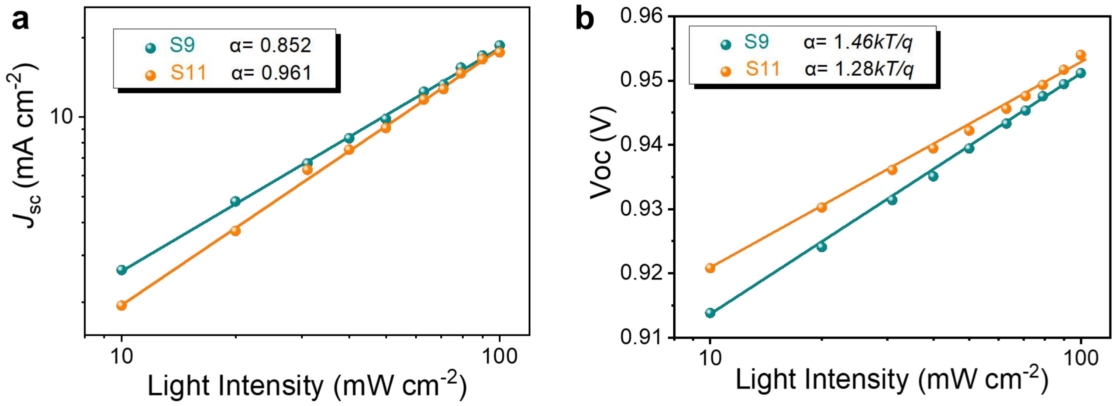

Supplementary Fig. 33 Dependence of **a** J_{sc} and **b** V_{oc} on different light intensity (P_{light}) of the OSCs devices.

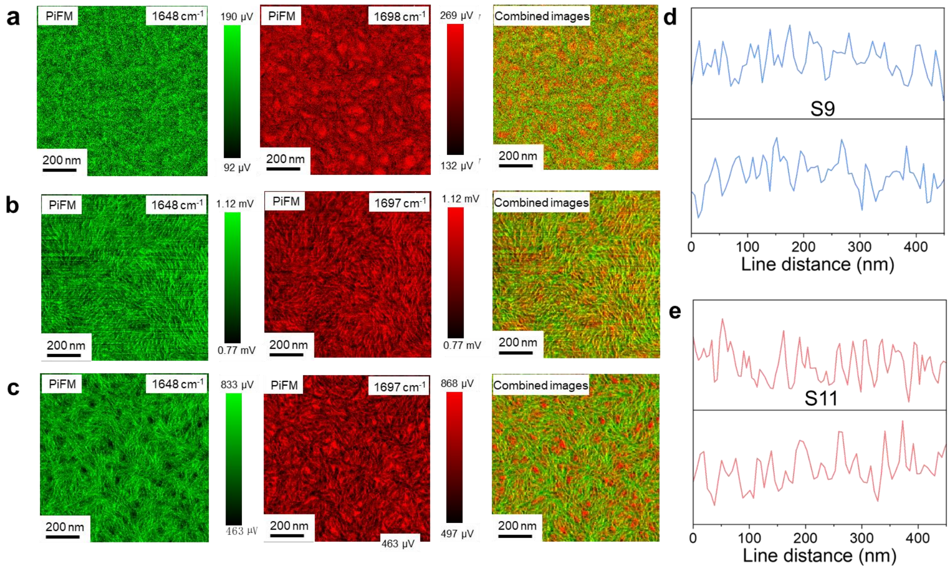

Supplementary Fig. 34 PiFM images of the films. The patterns and the combined images to provide chemical map of **a** PM6:L15, **b** S9 and **c** S11 system, respectively (the scale bar is 200 nm). The line profiles from the PiFM images are used to obtain the fibril width for **d** S9 and **e** S11 system. The fibril width is obtained from the full-width at half-maximum.

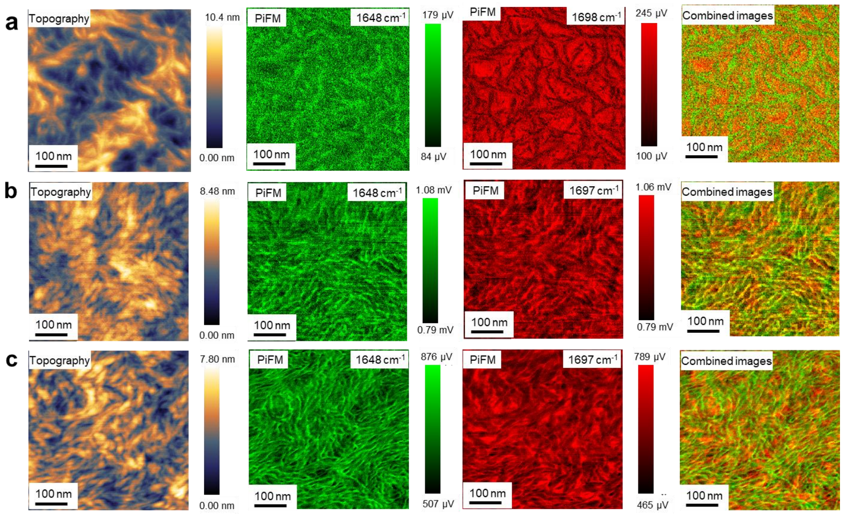

Supplementary Fig. 35 AFM topography and PiFM images of of **a** PM6:L15, **b** S9 and **c** S11 system, respectively (the scale bar is 100 nm).The patterns and the combined images were presented to provide chemical map for the corresponding films.

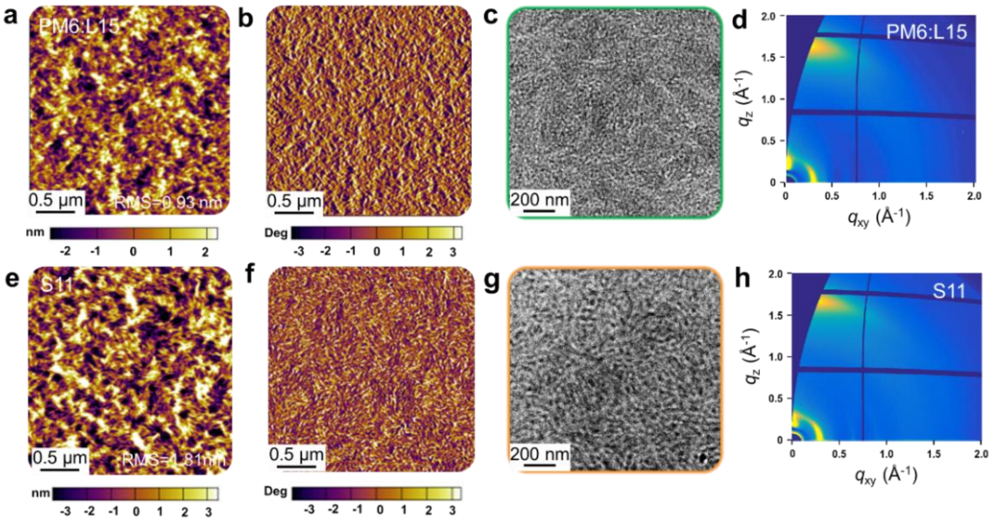

Supplementary Fig. 36 Morphological characteristics of the films. (a, e) AFM height, **(b, f)** phase, **(c, g)** TEM images and **(d, h)** 2D GIWAXS patterns of PM6:L15 and S11 films.

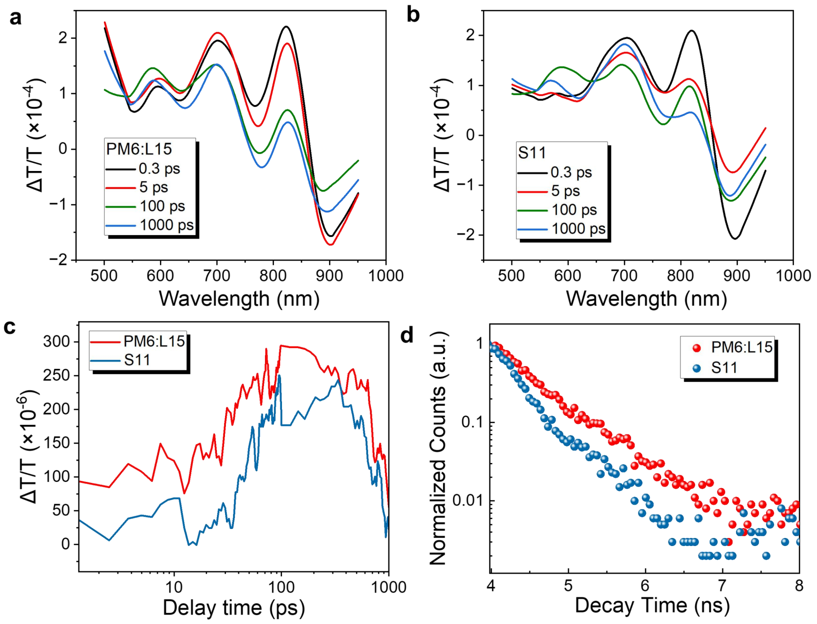

Supplementary Fig. 37 Charge transfer kinetic behaviors of OSCs. a Transient absorption spectra of the optimized PM6:L15 two-component system at indicated delay times. **b** Transient absorption spectra of the multicomponent S11 system at indicated delay times. **c** The hole transfer process in the optimized PM6:L15 and S11 films, respectively. **d** Normalized TRPL decay curves of PM6:L15 and d S11 films, respectively.

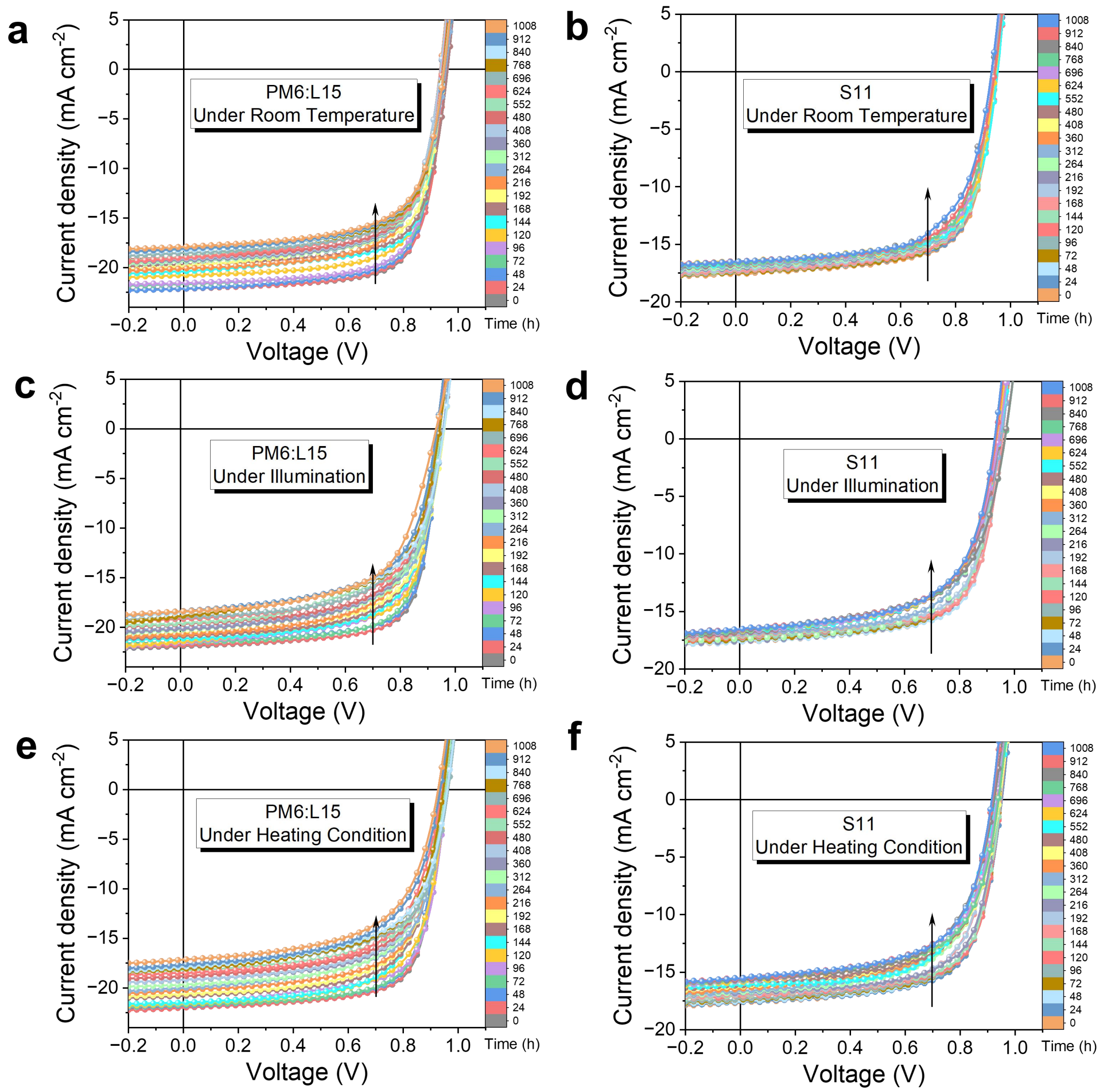

Supplementary Fig. 38 The changes of *J-V* characteristics of OSCs for up to 1008 h storage. a PM6:L15 and **b** S11 under room temperature, **c** PM6:L15 and **d** S11 under continuous illumination condition, **e** PM6:L15 and **f** S11 under 85 ℃ heated condition.

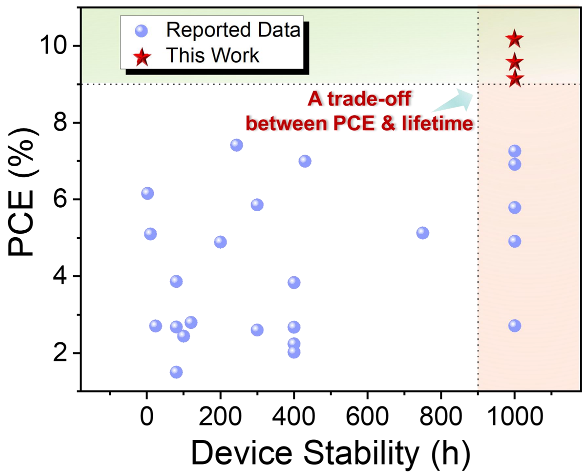

Supplementary Fig. 39 Plots of the PCE *versus* device lifetime for single-component OSCs and our multicomponent OSCs in this work.

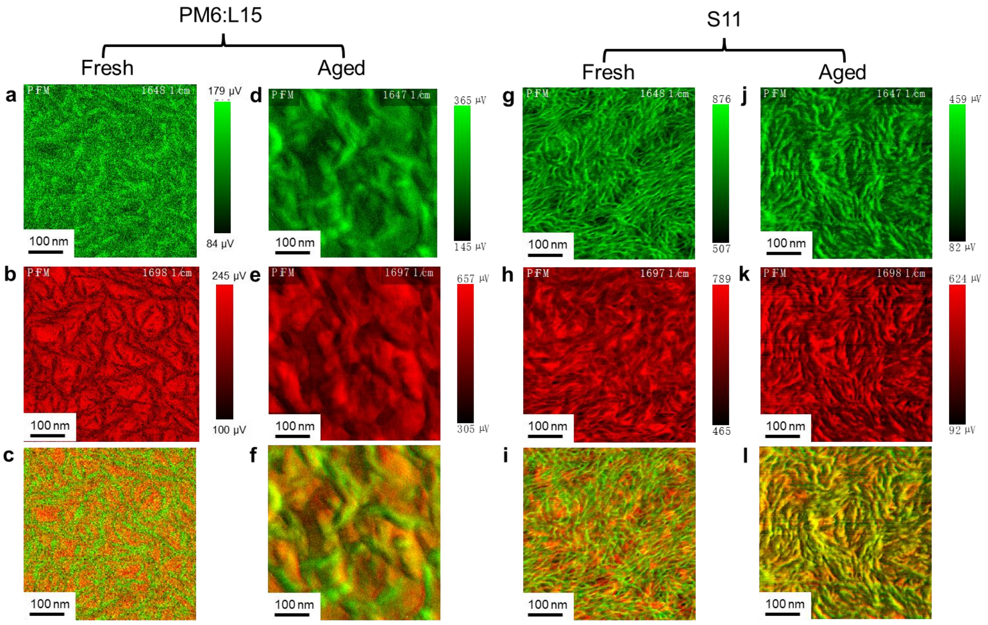

Supplementary Fig. 40 The OSCs based on PM6:L15 and S11 active layers under continuous heating of 716 h: morphology evolution of PiFM images (**a-c, g-i**) before and (**d-f, j-l**) after aging of PM6:L15 and S11 systems, respectively.

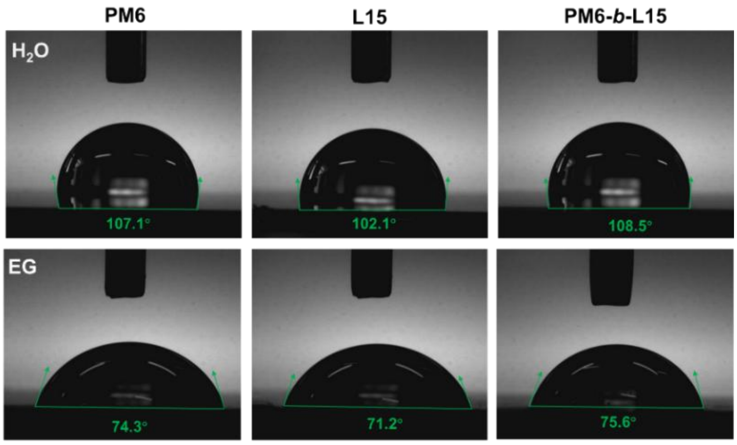

Supplementary Fig. 41 The photographs of water (H_2O) and ethylene glycol (EG) droplets on the top surfaces of PM6, L15 and PM6-*b*-L15 film. The contact angle measurement is conducted at the center of the substrates to avoid the edges of ITO anodes.

3. Supplementary Tables

Supplementary Table 1 Number-average molecular weight (M_n), weight-average molecular weight (M_w), Z-average molecular weight (M_z) and PDI of PM6, PM6-*b*-PY-IT, PM6-*b*-L15, S9 and S11.

Sample	M_n (kDa)	M_w (kDa)	M_z (kDa)	PDI
PM6	6.9	12.9	21.4	1.85
PM6-*b*-PY-IT	16.4	32.8	64.0	2.00
PM6-*b*-L15	18.6	57.2	133.1	3.07
S9	16.3	43.8	97.0	2.69
S11	18.6	50.4	83.9	2.71

Supplementary Table 2 The elemental analysis data of the photoactive materials.

Photoactive material	Element	Mass Fraction (%)	Mass Error (%)	Atomic Fraction (%)	Atomic Error (%)
PM6-*b*-PY-IT	N	70.46	4.29	76.72	3.75
	F	29.54	4.29	23.28	3.75
S9	N	72.90	3.68	77.97	3.10
	F	27.10	3.68	22.03	3.10
PM6-*b*-L15	N	72.83	4.45	79.06	3.94
	F	27.17	4.45	20.94	3.94
S11	N	75.25	3.36	80.31	2.78
	F	24.75	3.36	19.69	2.78

Supplementary Table 3 The elemental analysis data of PM6-*b*-L15 single component photoactive material.

Photoactive material	N (%)	C (%)	H (%)	S (%)
PM6-*b*-L15	2.67	69.76	7.08	15.69
	2.65	69.87	7.04	15.88

Supplementary Table 4 The mass content analysis data of S11, PM6-*b*-L15 and residual blocks.

Polymer	Mass content ratio of L15 block or segment in S11 (%)	Mass content ratio of PM6 block or segment in S11 (%)
S11	47.64	52.36
PM6-*b*-L15	42.35	50.42
Residual blocks	5.29	1.94

Supplementary Table 5 The basic properties of active materials.

Polymer	$E_g^{opt,a}$ (eV)	HOMO[b] (eV)	LUMO[b] (eV)
S9	1.39	-5.64	-3.68
S11	1.38	-5.71	-3.74

[a] $E_g^{opt}= 1240/\lambda_{onset}$.

[b] Calculated from cyclic voltammetry.

Supplementary Table 6 Number-average molecular weight (M_n), weight-average molecular weight (M_w), Z-average molecular weight (M_z) and PDI of different batches of S11.

Polymer	M_n (kDa)	M_w (kDa)	M_z (kDa)	PDI
S11-A1	18.6	50.4	83.9	2.71
S11-A2	14.2	30.7	50.0	2.16
S11-A3	11.3	25.3	41.9	2.24

Supplementary Table 7 The photovoltaic performance parameters of OSCs based on different batches of S11 under the illumination of AM 1.5G, 100 mw cm^{-2}.

Active Layer[a]	V_{oc} (V)	J_{sc} (mA cm^{-2})	Cal J_{sc} (mA cm^{-2})[b]	FF (%)	PCE_{max} (%)	PCE_{ave} (%)[c]
S11-A1	0.95	17.91	17.42	69.12	11.76	11.37
S11-A2	0.95	17.53	17.18	69.43	11.53	11.11
S11-A3	0.95	17.02	16.76	68.31	11.06	10.80

[a] The device area is 4.5 mm^2; [b] Integrated current density obtained from EQE spectra. [c]Average value from 15 devices.

Supplementary Table 8 Number-average molecular weight (M_n), weight-average molecular weight (M_w), Z-average molecular weight (M_z) and PDI of the PM6 blocks and S11.

Polymer	M_n (kDa)	M_w (kDa)	M_z (kDa)	PDI
PM6 1 h	4.9	7.0	9.7	1.42
PM6 2 h	6.9	12.9	21.4	1.85
PM6 3 h	10.8	16.9	24.0	1.58
S11-D1	26.8	82.8	161.1	3.09
S11-D2	18.6	50.4	83.9	2.71
S11-D3	16.1	42.3	112.3	2.63

Supplementary Table 9 The photovoltaic performance parameters of OSCs based on different batches of S11 under the illumination of AM 1.5G, 100 mw cm^{-2}.

Active Layer[a]	V_{oc} (V)	J_{sc} (mA cm^{-2})	Cal J_{sc} (mA cm^{-2})[b]	FF (%)	PCE_{max} (%)	PCE_{ave} (%)[c]
S11-D1	0.98	11.36	10.93	56.47	6.27	5.98
S11-D2	0.95	17.51	16.85	70.32	11.72	11.39
S11-D3	0.94	15.28	14.76	60.11	8.65	8.27

[a] The device area is 4.5 mm^2; [b] Integrated current density obtained from EQE spectra. [c]Average value from 15 devices.

Supplementary Table 10 Photovoltaic performance parameters of the OSCs incorporating PM6:L15, PM6-*b*-L15 and S11 photoactive layers under the illumination of AM 1.5G,100 mW cm^{-2}.

**Photoactive layer**[a]	V_{oc} **(V)**	J_{sc} **(mA cm^{-2})**	**FF (%)**	**PCE (%)**
PM6:L15	0.95	22.11	73.01	15.38
PM6-*b*-L15	0.95	21.05	71.59	14.31
S11	0.95	17.62	70.11	11.78

[a]The device area: 4.5 mm^2.

Supplementary Table 11 Top-gate/bottom-contact OTFT performance parameters of S9 and S11.

**Photoactive layer**	**Annealing (°C)**	$\mu_{h,OTFT}$ **(cm^2 V^{-1} s^{-1})**	$\mu_{e,OTFT}$ **(cm^2 V^{-1} s^{-1})**
S9	220	5.90×10^{-3}	8.65×10^{-4}
S11	250	1.43×10^{-2}	6.19×10^{-3}

Supplementary Table 12 Hole and electron mobilities for OSCs based on based on S9 and S11, respectively.

**Device**[a]	μ_h **($\times10^{-4}$ cm^2 V^{-1} s^{-1})**[b]	μ_e **($\times10^{-4}$ cm^2 V^{-1} s^{-1})**[b]	μ_h/μ_e
S9	1.93	0.59	3.27
S11	3.58	2.29	1.56

[a]The device area is 4.5 mm^2. [b]Average values with standard deviation were obtained from 20 devices.

Supplementary Table 13 G_{max} parameters of OSCs based on S9 and S11, respectively.

Active Layer	J_{sat} (mA cm^{-2})	L (nm)	G_{max} (m^{-3} s^{-1})
S9	21.58	116	1.16×10^{28}
S11	19.42	101	1.20×10^{28}

Supplementary Table 14 The parameters of exciton dissociation and charge collection efficiency based on S9 and S11, respectively.

Active Layer	J_{ph} [a] (mA cm^{-2})	J_{ph} [b] (mA cm^{-2})	J_{sat} (mA cm^{-2})	P_{diss} (%)	P_{coll} (%)
S9	18.70	16.26	21.74	86.01	74.79
S11	17.59	15.93	19.15	91.85	83.18

[a] Under short circuit condition. [b] Under the maximal power output condition.

Supplementary Table 15 Summary of IP (100) and OOP (010) coherence lengths of the S9 and S11 films.

Sample	d-$_{(100)}$^{IP} (Å^{-1})	$L_{C(100)}$^{IP} (nm)	d-$_{(010)}$^{OOP} (Å^{-1})	$L_{C(010)}$^{OOP} (nm)
S9	21.8	19.9	3.61	2.83
S11	21.6	20.4	3.61	3.85

Supplementary Table 16 Summary of IP (100) and OOP (010) coherence lengths of the PM6:L15 and S11 films.

Sample	d-$_{(100)}$^{IP} (Å^{-1})	$L_{C(100)}$^{IP} (nm)	d-$_{(010)}$^{OOP} (Å^{-1})	$L_{C(010)}$^{OOP} (nm)
PM6:L15	21.5	21.2	3.61	4.27
S11	21.6	20.4	3.61	3.85

Supplementary Table 17 Normalized photovoltaic parameters of optimized PM6:L15 based OSCs in nitrogen atmosphere for up to 1008 h storage under room temperature (RT).

Time (h)	V_{oc} (V)	J_{sc} (mA cm^{-2})	FF (%)	PCE (%)
0	100.00	100.00	100.00	100.00
6	100.87	100.18	98.03	99.07
12	99.97	100.08	97.62	97.67
24	100.08	97.92	97.54	95.59
36	99.49	96.11	97.37	93.11
48	99.81	93.94	97.10	91.01
60	100.85	92.14	95.83	89.04
72	101.09	88.96	96.41	86.70
90	100.47	89.65	93.60	84.31
114	98.87	90.86	91.87	82.54
138	100.91	87.50	91.68	80.95
162	100.26	87.44	91.20	79.96
186	99.04	86.85	92.14	79.26
210	99.01	86.22	92.15	78.66
234	100.35	85.83	91.31	78.65
258	100.27	85.54	91.28	78.30
282	99.74	85.90	90.14	77.23
354	100.34	83.73	90.90	76.37
426	100.05	82.25	91.42	75.23
498	100.25	82.34	89.58	73.94
618	100.59	81.86	89.28	73.52
1008	100.41	80.95	89.74	72.94

Supplementary Table 18 Normalized photovoltaic parameters of optimized S11-based OSCs in nitrogen atmosphere for up to 1008 h storage under room temperature (RT).

**Time (h)**	V_{oc} **(V)**	J_{sc} **(mA cm^{-2})**	**FF (%)**	**PCE (%)**
0	100.00	100.00	100.00	100.00
6	100.31	99.50	99.80	99.60
12	100.31	99.39	99.44	99.13
24	99.25	99.22	99.13	97.62
36	99.25	98.93	98.96	97.17
48	100.43	98.82	98.86	98.10
60	100.31	98.65	98.51	97.48
72	99.72	98.28	98.36	96.40
90	99.25	97.79	98.28	95.39
114	100.31	97.05	98.14	95.53
138	100.31	96.93	97.88	95.17
162	100.31	96.19	97.66	94.23
186	100.31	95.68	97.46	93.54
210	100.31	95.45	97.33	93.19
234	99.25	95.34	97.05	91.83
258	100.31	95.17	96.80	92.40
282	100.31	95.22	96.27	91.95
354	99.09	94.77	95.54	89.72
426	99.25	94.67	95.24	89.49
498	99.39	94.54	95.00	89.27
618	99.92	94.30	94.68	89.22
1008	99.57	94.08	94.50	88.52

Supplementary Table 19 Normalized photovoltaic parameters of optimized PM6:L15 based OSCs in nitrogen atmosphere for up to 1008 h storage under continuous illumination condition.

Time (h)	V_{oc} (V)	J_{sc} (mA cm^{-2})	FF (%)	PCE (%)
0	100.00	100.00	100.00	100.00
6	99.97	99.46	99.63	99.06
12	100.24	98.14	99.33	97.71
24	100.66	97.69	97.67	96.00
36	99.19	98.14	95.57	93.03
48	100.00	97.82	93.76	91.71
60	100.09	96.42	91.42	88.23
72	101.34	95.36	89.43	86.42
90	101.75	94.05	88.77	84.95
114	100.03	94.58	86.80	82.13
138	100.64	91.45	88.21	81.19
162	99.84	92.35	87.67	80.83
186	100.01	91.86	86.85	79.79
210	99.96	89.58	87.85	78.67
234	99.86	89.72	87.12	78.06
258	100.19	89.34	85.61	76.63
282	99.71	87.39	86.17	75.08
354	99.78	86.63	85.27	73.70
426	99.87	86.14	84.02	72.28
498	101.59	84.52	83.54	71.73
618	99.92	84.07	84.68	71.13
1008	99.98	83.98	83.61	70.21

Supplementary Table 20 Normalized photovoltaic parameters of optimized S11-based OSCs in nitrogen atmosphere for up to 1008 h storage under continuous illumination condition.

Time (h)	V_{oc} (V)	J_{sc} (mA cm^{-2})	FF (%)	PCE (%)
0	100.00	100.00	100.00	99.98
6	100.00	99.72	100.19	99.89
12	100.00	99.04	100.09	99.10
24	100.00	98.75	99.59	98.33
36	99.23	98.36	99.66	97.25
48	100.00	97.84	99.05	96.89
60	99.31	97.17	98.64	95.17
72	100.41	96.67	98.43	95.52
90	98.95	96.08	97.69	92.86
114	98.57	95.97	95.69	90.51
138	98.25	95.39	94.36	88.42
162	98.95	95.63	92.26	87.28
186	98.95	94.95	91.34	85.79
210	98.16	95.41	90.99	85.20
234	99.48	95.11	90.08	85.22
258	98.95	94.78	89.49	83.91
282	98.73	94.71	89.97	84.11
354	99.34	94.66	89.02	83.69
426	100.00	94.38	88.44	83.45
498	101.05	94.32	88.61	84.45
618	100.13	94.18	88.79	83.71
1008	99.64	94.01	88.54	82.93

Supplementary Table 21 Normalized photovoltaic parameters of optimized PM6:L15 based OSCs in nitrogen atmosphere for up to 1008 h storage under 85 °C heated condition.

**Time (h)**	V_{oc} **(V)**	J_{sc} **(mA cm^{-2})**	**FF (%)**	**PCE (%)**
0	100.00	100.00	100.00	100.00
6	99.70	100.02	99.43	99.16
12	100.11	98.79	98.79	97.70
24	100.33	98.69	97.57	96.61
36	101.43	98.22	95.25	94.89
48	101.34	98.13	93.61	93.09
60	99.26	97.72	92.97	90.18
72	100.04	94.63	92.30	87.38
90	100.21	93.73	91.66	86.09
114	100.48	92.02	90.69	83.86
138	100.72	90.96	90.03	82.49
162	100.83	89.43	89.40	80.61
186	100.77	87.40	88.96	78.35
210	100.02	87.19	88.53	77.21
234	100.16	85.64	87.93	75.42
258	100.85	82.93	87.45	73.14
282	99.66	83.98	86.48	72.38
354	102.10	81.68	85.63	71.40
426	100.21	82.16	84.42	69.50
498	101.73	81.31	82.50	68.25
618	100.09	79.76	82.54	65.90
1008	102.77	78.17	81.82	65.73

Supplementary Table 22 Normalized photovoltaic parameters of optimized S11 based OSCs in nitrogen atmosphere for up to 1008 h storage under 85 ℃ heated condition.

Time (h)	V_{oc} (V)	J_{sc} (mA cm^{-2})	FF (%)	PCE (%)
0	100.00	100.00	100.00	100.03
6	100.00	99.72	99.69	99.44
12	98.95	99.38	99.49	97.86
24	100.00	98.07	99.09	97.21
36	98.95	97.68	98.74	95.46
48	100.00	96.89	98.26	95.23
60	100.00	96.32	97.76	94.19
72	99.27	96.19	96.92	92.56
90	98.95	95.47	96.69	91.36
114	98.84	95.25	95.92	90.33
138	98.95	94.62	96.04	89.95
162	98.95	93.94	93.87	87.28
186	98.76	93.17	93.78	86.32
210	99.48	92.10	93.65	85.83
234	98.76	91.61	92.88	84.05
258	97.51	91.00	92.55	82.15
282	98.92	89.65	92.31	81.89
354	98.67	89.36	92.04	81.18
426	99.04	88.82	91.86	80.84
498	98.95	88.39	91.72	80.24
618	99.09	88.26	91.53	80.07
1008	99.51	88.01	91.36	80.04

Supplementary Table 23 Investigations of the contact angles, surface energy and interfacial tension values of PM6, L15 and PM6-*b*-L15.

Polymers	Contact		Surface Energy [mJ m^{-2}]	Relative χ^{α}
	Water	Ethylene glycol		
PM6	107.1°	74.3°	30.6	0.11K
L15	102.1°	71.2°	28.4	0.03k
PM6-*b*-L15	108.5°	75.6°	26.6	/

$\chi^{\alpha} = K(\sqrt{\gamma(polymer)} - (\sqrt{\gamma(PM6\text{-}b\text{-L15})}\,)^2$, where γ is the surface tension and K is a constant.

4. Supplementary References

1. Shi, Y. *et al.* Distannylated Bithiophene Imide: Enabling High-Performance n-Type Polymer Semiconductors with an Acceptor-Acceptor Backbone. *Angew. Chem.* **132**, 14557-14565 (2020).
2. Luo, Z. *et al.* Precisely controlling the position of bromine on the end group enables well-regular polymer acceptors for all-polymer solar cells with efficiencies over 15%. *Adv. Mater.* **32**, 2005942 (2020).
3. Sun, H. *et al.* Regioregular narrow-bandgap n-type polymers with high electron mobility enabling highly efficient all-polymer solar cells. *Adv. Mater.* **33**, 2102635 (2021).
4. Ku, S.-Y. *et al.* A modular strategy for fully conjugated donor–acceptor block copolymers. *J. Am. Chem. Soc.* **134**, 16040-16046 (2012).
5. Li, S. *et al.* Narrow-Bandgap Single-Component Polymer Solar Cells with Approaching 9% Efficiency. *Adv. Mater.* **33**, 2101295 (2021).
6. Wu, Y. *et al.* A conjugated donor-acceptor block copolymer enables over 11% efficiency for single-component polymer solar cells. *Joule* **5**, 1800-1815 (2021).
7. Yang, X. *et al.* Over 13% Efficient Single-Component Organic Solar Cells Enabled by Adjusting the Conjugated-Length of Intermediate PBDB-T Block. *Adv. Funct. Mater.* 2208412 (2022).
8. Wang, Y. *et al.* (Semi) ladder-type bithiophene imide-based all-acceptor semiconductors: Synthesis, structure–property correlations, and unipolar n-type transistor performance. *J. Am. Chem. Soc.* **140**, 6095-6108 (2018).
9. Heeger, A. J. 25th anniversary article: bulk heterojunction solar cells: understanding the mechanism of operation. *Adv. Mater.* **26**, 10-28 (2014).
10. Zhong, Y. *et al.* Molecular helices as electron acceptors in high-performance bulk heterojunction solar cells. *Nat. Commun.* **6**, 8242 (2015).
11. Qiu, B. *et al.* Highly Efficient All-Small-Molecule Organic Solar Cells with Appropriate Active Layer Morphology by Side Chain Engineering of Donor Molecules and Thermal Annealing. *Adv. Mater.* **32**, 1908373 (2020).
