# Supplementary material for: Efficient and stable organic solar cells enabled by multicomponent photoactive layer based on one-pot polymerization
Source: Nat Commun. 2023 Feb 21;14:967. doi: 10.1038/s41467-023-36413-3 (PMC9944902; doi:10.1038/s41467-023-36413-3)
Supplement: Supplementary file 2 — Solar Cells Reporting Summary [file 41467_2023_36413_MOESM2_ESM.pdf]

nature research

Corresponding author(s): Xugang Guo

Solar Cells Reporting Summary

Nature Research wishes to improve the reproducibility of the work that we publish. This form is intended for publication with all accepted papers reporting the characterization of photovoltaic devices and provides structure for consistency and transparency in reporting. Some list items might not apply to an individual manuscript, but all fields must be completed for clarity.

For further information on Nature Research policies, including our data availability policy, see Authors & Referees.

Experimental design

Please check: are the following details reported in the manuscript?

1. Dimensions

Item	Yes/No	Response
Area of the tested solar cells	☒ Yes ☐ No	The active area of the standard solar cell fabricated in our lab is 0.045 cm2.
Method used to determine the device area	☒ Yes ☐ No	The device area was calculated by multiplying the width of ITO electrode and Ag electrode.

2. Current-voltage characterization

Item	Yes/No	Response
Current density-voltage (J-V) plots in both forward and backward direction	☐ Yes ☒ No	The J-V curves for the organic solar cells were only scanned in forward direction.
Voltage scan conditions<br>*For instance: scan direction, speed, dwell times*	☒ Yes ☐ No	From -0.3 V to 1.2 V with 0.01 V interval, 1ms delay.
Test environment<br>*For instance: characterization temperature, in air or in glove box*	☒ Yes ☐ No	The devices were characterized in nitrogen-filled glove box at room temperature.
Protocol for preconditioning of the device before its characterization	☐ Yes ☒ No	No preconditioning protocol.
Stability of the J-V characteristic<br>*Verified with time evolution of the maximum power point or with the photocurrent at maximum power point; see ref. 7 for details.*	☒ Yes ☐ No	We measured the shelf-life, light and thermal stability of PM6:L15 and S11-based devices for up to 1008 h, as shown in Figure 4.

3. Hysteresis or any other unusual behaviour

Item	Yes/No	Response
Description of the unusual behaviour observed during the characterization	☐ Yes ☒ No	No hysteresis was observed in our devices.
Related experimental data	☐ Yes ☒ No	No unusual behavior was found for the related experimental data.

4. Efficiency

Item	Yes/No	Response
External quantum efficiency (EQE) or incident photons to current efficiency (IPCE)	☒ Yes ☐ No	The EQE curves were shown in Figure 2c.
A comparison between the integrated response under the standard reference spectrum and the response measure under the simulator	☒ Yes ☐ No	The relative information was provided in Table 1. The integrated Jsc obtained from the EQE spectra agreed well with the Jsc value measured from the J–V curves (witnin 5% deviation).
For tandem solar cells, the bias illumination and bias voltage used for each subcell	☐ Yes ☒ No	We did not fabricate the tandem solar cells in this study.

5. Calibration

Item	Yes/No	Response
Light source and reference cell or sensor used for the characterization	☒ Yes ☐ No	All current-voltage (J-V) characteristics of the devices were measured under simulated AM 1.5G irradiation (100 mW cm-2) using a Xe lamp-based SS-F5-3A Solar Simulator (Enli Technology, Inc.). A Xe lamp equipped with an AM1.5G filter was used as the white light source. The light intensity was calibrated with a standard single-crystal Si solar cell.

Confirmation that the reference cell was calibrated and certified

☒ Yes ☐ No

The standard monocrystalline silicon reference cell was made by Enli Technology, CO., Ltd., and was calibrated by the company.

Calculation of spectral mismatch between the reference cell and the devices under test

☐ Yes ☒ No

Spectral mismatch factor was not considered.

6. Mask/aperture

Size of the mask/aperture used during testing

☐ Yes ☒ No

We didn't use masks during testing in the lab.

Variation of the measured short-circuit current density with the mask/aperture area

☐ Yes ☒ No

We didn't use masks during testing in the lab.

7. Performance certification

Identity of the independent certification laboratory that confirmed the photovoltaic performance

☐ Yes ☒ No

We didn't do the independent certification. Because the device efficiency achieved in this case is not so high. In addition, our work is mainly focus on the stability issue. The performance certification is not very important in this work.

A copy of any certificate(s)
Provide in Supplementary Information

☐ Yes ☒ No

We didn't do the performance certification via the independent certification laboratory.

8. Statistics

Number of solar cells tested

☒ Yes ☐ No

Data in parentheses are average values with standard deviation from 15 devices.

Statistical analysis of the device performance

☒ Yes ☐ No

Statistical results of the devices are shown in Table 1.

9. Long-term stability analysis

Type of analysis, bias conditions and environmental conditions
For instance: illumination type, temperature, atmosphere humidity, encapsulation method, preconditioning temperature

☒ Yes ☐ No

Long-term stability analysis and corresponding environmental conditions can be found in Figure 4.
